# Dissecting the regulation rules of cancer-related miRNAs based on network analysis

**DOI:** 10.1038/srep34172

**Published:** 2016-10-03

**Authors:** Zhongyu Liu, Yanzhi Guo, Xuemei Pu, Menglong Li

**Affiliations:** 1 College of Life Science, Sichuan University, Chengdu, Sichuan 610064, PR China; 2 College of Chemistry, Sichuan University, Chengdu, Sichuan 610064, PR China

## Abstract

miRNAs (microRNAs) are a set of endogenous and small non-coding RNAs which specifically induce degradation of target mRNAs or inhibit protein translation to control gene expression. Obviously, aberrant miRNA expression in human cells will lead to a serious of changes in protein-protein interaction network (PPIN), thus to activate or inactivate some pathways related to various diseases, especially carcinogenesis. In this study, we systematically constructed the miRNA-regulated co-expressed protein-protein interaction network (CePPIN) for 17 cancers firstly. We investigated the topological parameters and functional annotation for the proteins in CePPIN, especially for those miRNA targets. We found that targets regulated by more miRNAs tend to play a more important role in the forming process of cancers. We further elucidated the miRNA regulation rules in PPIN from a more systematical perspective. By GO and KEGG pathway analysis, miRNA targets are involved in various cellular processes mostly related to cell cycle, such as cell proliferation, growth, differentiation, etc. Through the Pfam classification, we found that miRNAs belonging to the same family tend to have targets from the same family which displays the synergistic function of these miRNAs. Finally, the case study on miR-519d and miR-21-regulated sub-network was performed to support our findings.

MiRNAs, also known as microRNAs, are a subgroup of small non-coding RNAs in eukaryotic cells. Mature miRNAs are ~22nt long and single stranded RNA molecules processed from hairpin-like precursors (pre-miRNAs)^1^. MiRNAs have a highly conserved region called “seed sequence” which is of 2–8nt in length in their 5′ end. It has been reported that the “seed sequence” region plays an important role in specifically recognizing and binding mRNAs in miRNA regulation^2^. After binding, miRNAs tend to degrade mRNAs or inhibit their translation at post-transcription level^3^,^4^. Most products of these mRNAs are essential proteins such as signaling proteins, enzymes and transcription factors (TFs) that are involved in various cellular processes. Most of these proteins have been proved to be hub or bottleneck proteins in human protein-protein interaction network (PPIN)^5^. In this case, aberrant miRNA regulation would lead to a dynamic change in human PPIN, thus to activate or inhibit some signaling pathways related to diseases even cancers^6^,^7^. So exploiting miRNAs for cancer diagnosis, prognosis and therapeutics has a promising future in clinical medicine^8^,^9^.

In the last few years, the studies on miRNA-regulated human PPIN have drawn much attention and have been applied to cancer researches. Liang and Li^10^ have revealed that protein connectivity in human PPIN is positively correlated with the number of miRNA target-site types in the 3′ untranslated region (3′ UTR) of mRNA encoding the protein. Hsu *et al*.^11^ extended Liang and Li’s work and named the direct targets of miRNAs “L0 proteins” and the interacting partners of these targets “L1 proteins”. They also compared the four important topological parameters, namely degree, betweenness centrality, clustering coefficient and closeness centrality of L0 proteins and L1 proteins jointly with random selected proteins, which revealed that miRNA-regulated targets and their interacting partners jointly show significantly higher connectivity and modularity than random selected proteins, even than direct targets alone. In their following work^12^, they revealed that L0 proteins and L1 proteins together also show a stronger functional relationship than L0 proteins alone. They further named the network formed by L0 proteins and L1 proteins the co-expressed protein-protein interaction network (CePPIN) which can also be deemed as miRNA-regulated CePPIN. Recently, integrating miRNA targets and PPIN makes it more efficient to dissect important miRNAs and proteins, especially for various diseases including cancers^13^,^14^,^15^,^16^,^17^. Furthermore, the topological properties and functional annotation of miRNA targets in human PPIN are used as the effective characters to predict and identify new biomarkers^18^ also drug targets for relevant diseases^19^. So combining expression profile and network analysis will improve the prediction precision and give a better understanding of the interaction mechanisms between these molecules^20^,^21^. However, the existing researches concentrate on only one kind of cancer, so whether these findings are applicable to other cancers still remains unsolved. That is to say, there is a lack of general conclusions for most cancers to the best of our knowledge.

In this study, we firstly constructed the miRNA-regulated CePPIN for totally 17 cancers which have been the key cancer types so far. Here we used topological parameters and functional annotation to investigate the rules of miRNA regulation in human PPIN. We found that degrees, betweenness centralities and closeness centralities of miRNA targets are higher than non-targets and positively correlated with the number of miRNAs regulating them. Moreover, the functions of targets regulated by more miRNAs indicate to be more similar and they are all common factors closely related to various cancers, such as cell proliferation, apoptosis, transcription and diverse cancer-related pathways. Some members of the same miRNA family also show obvious functional similarity by targeting proteins of the same family, especially some miRNAs of mir-10 family, mir-17 family and mir-34 family respectively from our analysis results. Finally two representative miRNAs were selected, including miR-519d that regulates hepatocellular carcinoma only in our dataset and miR-21 that regulates 12 cancers including hepatocellular carcinoma in our dataset. Their sub-network were constructed and functional analysis were implemented to stress our findings more concretely.

## Results

### Construction of miRNA-regulated CePPIN for 17 cancers

We selected 17 representative cancers which have the most numbers of miRNAs from the original dataset. All these cancers are widely studied in recent researches, namely acute myeloid leukemia, bladder cancer, breast cancer, colon cancer, colorectal cancer, esophageal squamous cell carcinoma, gastric cancer, glioblastoma, glioma, hepatocellular carcinoma, lung cancer, melanoma, neuroblastoma, non-small cell lung cancer, ovarian cancer, pancreatic cancer and prostate cancer. Totally, 288 different miRNAs are involved and all 573 miRNAs targets are collected to induce the 17 cancers.

**Figure 1:**
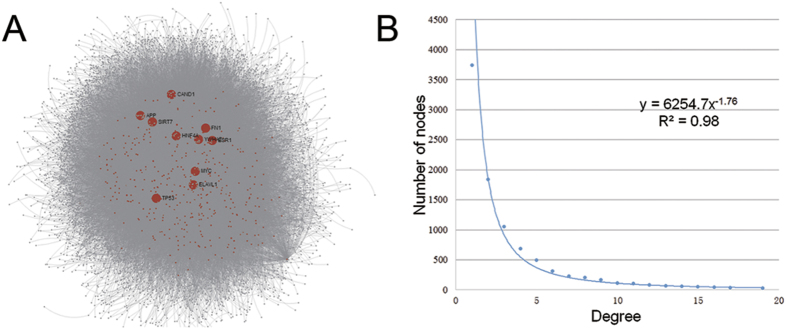
The visualization of CePPIN. (**A**) MiRNA-regulated CePPIN generated by Network Analyst. Red nodes are miRNA targets and grey nodes are non-targets. The 10 nodes in bigger size and with labels have the highest degrees in CePPIN. (**B**) Degree distribution of proteins in CePPIN.

We used the 573 targets as source to compile the human protein-protein interactions (PPIs) information from InnateDB^22^. Overall, we obtained 9782 proteins (552 targets and 9230 co-expressed proteins) and 36712 PPIs as the original dataset of CePPIN. 21 targets were discarded for lacking the interactome information. The visualization of CePPIN is shown in Fig. 1A. The degree distribution of CePPIN is shown in Fig. 1B and the average shortest path length of all the nodes is about 3.16. We can see that CePPIN presents the properties of small-world and scale-free network, which indicates that a small number of proteins called hubs directly regulate most of other members in the network, so the network is more robust against random variation but more fragile against some aberration of the hubs compared with random network. In Fig. 1A, the bigger red nodes with labels are top ten-ranked proteins by their degrees in CePPIN. Their degrees are all over 600 and they are all miRNA targets. More information of the ten proteins is also shown in Table 1. It can also be seen explicitly that the overwhelming majority of miRNA targets locate centrally and may play an important role in CePPIN.

### Statistical analysis of miRNAs and targets

**Figure 2:**
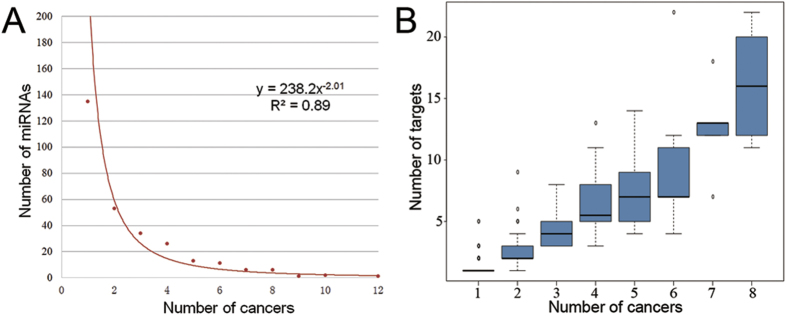
Statistical results of cancers, miRNAs and targets. (**A**) The relationship between the number of cancers and the number of miRNAs regulating a certain amount of cancers. (**B**) The relationship between the number of cancers miRNAs regulate and the number of targets these miRNAs have.

For 17 cancers and 288 miRNAs, we counted the number of cancers that each miRNA regulates. We found that almost a half of the 288 miRNAs regulate only one cancer and the number of miRNAs would decrease with the increasing of their regulating cancers (Fig. 2A). Moreover, only 4 miRNAs are involved in 9 or more cancers. They are miR-203 (9 cancers), miR-34a (10 cancers), miR-145 (10 cancers) and mir-21 (12 cancers) respectively and they all have been reported to be the common miRNAs in various cancers and other human diseases. For example, miR-21 has been early validated by experiments *in vitro* to regulate the antiapoptotic protein Bcl-2 and it functions as an oncogene^23^. MiR-34a has been reported to induce apoptosis by directly down-regulating Bcl-2 and some cyclin also CDK proteins so that it functions as a tumor suppressor^24^.

For 288 miRNAs and 573 targets, we counted the number of targets for each miRNA. Then, we compared the number of cancers and the number of targets for each miRNA. We found that miRNAs involved in more cancers tend to have more targets (Fig. 2B), which indicates that they may have more abundant functions and regulate multiple signaling pathways to induce carcinogenesis.

### Topological properties for miRNA-regulated CePPIN

**Figure 3:**
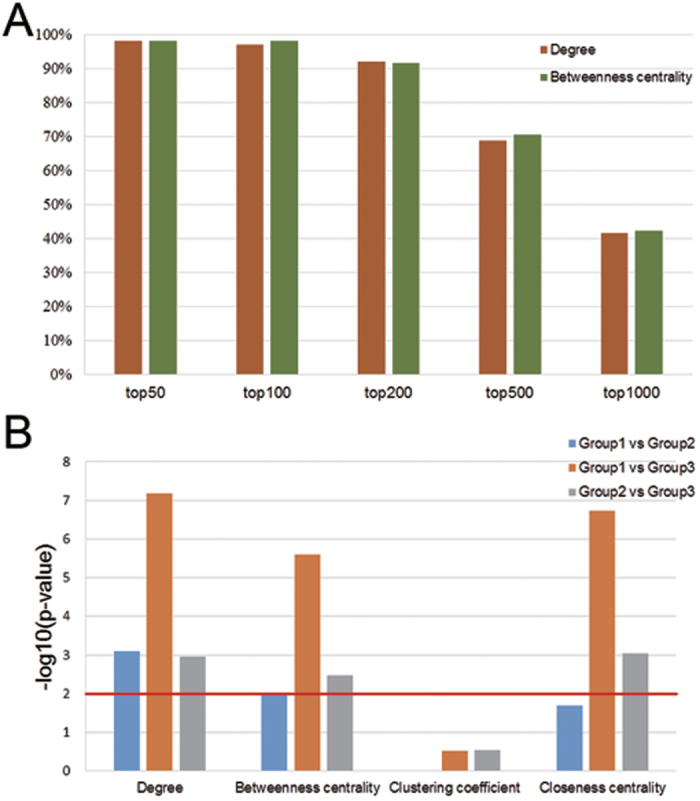
Topological properties of miRNA-regulated CePPIN. (**A**) The ratios of miRNA targets in top 50, 100, 200, 500, 1000 nodes sorted by degree and betweenness centrality respectively. (**B**) The Wilcoxon rank sum test results of the differences of topological parameters between each pairs in 3 groups of miRNA targets. Y axis represents the -log10(p-value). The value higher than 2 (p-value < 0.01) means that the difference of a certain parameter between the two groups of targets is significant.

We computed the four main topological parameters of all the nodes in CePPIN, namely degree, betweenness centrality, clustering coefficient and closeness centrality. We found the average degree, betweenness centrality and closeness centrality of miRNA targets are much higher than those of all the nodes in CePPIN. At the same time, clustering coefficients of miRNA targets are much lower (Table 2). To further investigate the role of miRNA targets in CePPIN, we counted the percentage of targets in top 50, 100, 200, 500 and 1000-ranked nodes by their degrees and betweenness centralities respectively in the whole CePPIN. We found that most of the targets have the highest degree and betweenness centrality in CePPIN (Fig. 3A and Supplementary Table S1).

Moreover, to reveal the relationship between the four topological parameters of miRNA targets in CePPIN and the number of miRNAs regulating them, we divided these targets into 3 groups according to the number of miRNAs regulating them. Group 1 contains 356 targets specifically regulated by only one miRNA; Group 2 contains 154 targets regulated by 2~4 miRNAs; Group 3 contains 42 targets widely regulated by 5 or more miRNAs. Then, we used Wilcoxon rank sum test for pairwise comparison on the four topological parameters of the targets in each group. The results (Fig. 3B) show that from Group 1 to Group 3, the degrees and betweenness centralities of targets grow significantly larger with the increase of the number of miRNAs regulating them. Moreover, the closeness centralities of targets in Group 3 are significantly higher than those in Group 1 and Group 2. From Table 3, the average closeness centrality of targets in Group 2 is higher than that in Group 1, although it shows no significant difference from rank sum test (Fig. 3B). Yet, the average clustering coefficient of targets in Group 2 and Group 3 is lower than that in Group 1 but it also shows no significance either.

### Functional analysis of miRNA targets in CePPIN

**Figure 4:**
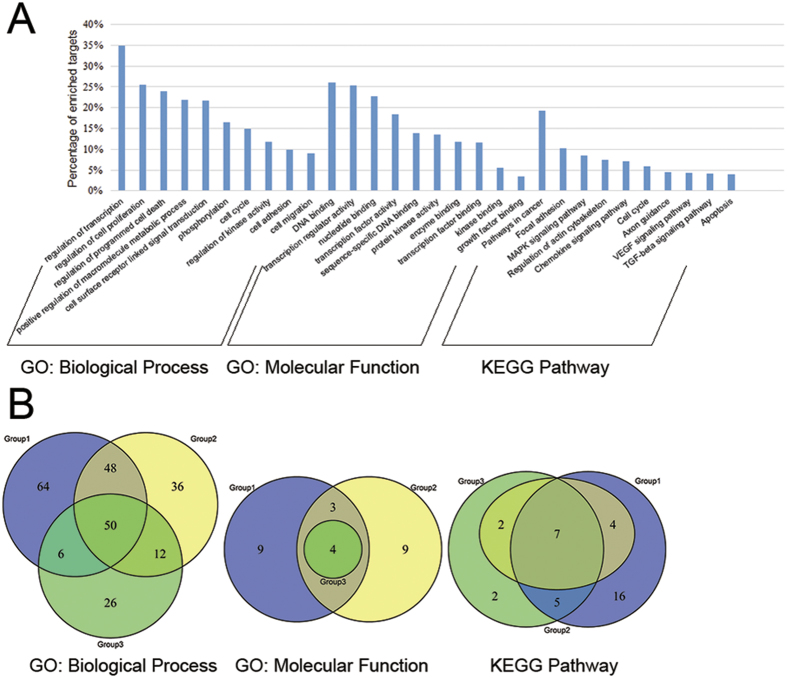
Functional analysis of miRNA targets in CePPIN. (**A**) The selected most enriched GO: biological process, GO: molecular function and KEGG pathway terms (p-value < 0.05 and FDR < 0.05) for all miRNA targets. (**B**) The number and overlaps of GO: biological process, GO: molecular function and KEGG pathway terms (p-value < 0.05 and FDR < 0.05) for miRNA targets in 3 group respectively. The blue, yellow and green circles indicate the terms of Group 1, Group 2 and Group 3 respectively.

We performed gene ontology (GO) and KEGG pathway analysis on 573 miRNA targets. Totally, 420 GO: biological process (GO: bp), 44 GO: molecular function (GO: mf) and 42 KEGG pathway terms (p-value < 0.05 and FDR < 0.05) were found for these targets. Figure 4A shows the selected top enriched pathway terms for these targets. We can see that the most enriched biological process term is “GO: 0045449~regulation of transcription”, “GO: 0042127~regulation of cell proliferation” and “GO: 0043067~regulation of programmed cell death”, all of which relate tightly with cell cycle to induce malignant proliferation and growth of tumor cells. Some terms about phosphorylation and kinase activity can be seen among the main regulation approaches of cell cycle. Cell adhesion and migration is relative to metastasis during cancer progression. All the molecular function terms are about the binding of DNA, transcription factor, kinase and growth factor, which are all molecular mechanisms related to cell cycle. As for KEGG pathway, besides the pathways for a specific caner like “hsa05215: Prostate cancer”, some cancer-related pathways such as MAPK, TGFβ and VEGF signaling pathways are among the top enriched pathway terms. Moreover, some pathways related to cell cycle such as “hsa04210: Apoptosis” and focal adhesion are also included. There are also some immune-related pathways such as chemokine, B cell receptor and Toll-like receptor signaling pathways in our results, of which the latter two pathways are shown in Supplementary Table S2. The activation of these pathways can be a mechanism for normal cells responding to the cancer cells invasion.

Afterwards, we imported targets in Group 1, Group 2 and Group 3 into DAVID respectively to get the functional information for each group. Then we investigated the common GO: bp, GO: mf and KEGG pathway terms among all 3 groups (Supplementary Table S3) and the exclusive terms of each group, which is displayed in Fig. 4B. We can observe that, from Group 1 to Group 3, the targets show a higher functional similarity with more miRNA regulating them and the targets regulated by the most miRNAs tend to have the most enriched function terms of all the targets, especially viewed from the molecular function and KEGG pathway information.

**Figure 5:**
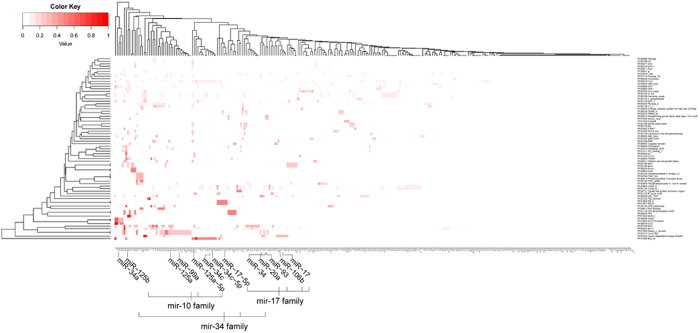
Heat map of the regulation relationship between miRNAs and protein families. Deeper color of the lump in heat map indicates a higher ratio between targets owned by corresponding miRNA and all the targets in corresponding Pfam term. Some miRNA members in mir-10, mir-17 and mir-34 family is shown in a zoom-in view.

To further study the relationship between miRNAs and the function of the targets and then figure out the regulation preference of miRNAs belonging to the same family, we categorized these targets in Pfam to get family information. After removing the redundant terms, we totally got 73 Pfam terms which contains family information of 287 targets, but some targets were categorized into more than one Pfam term. Then, we computed the ratio between targets of each miRNA categorized into a Pfam term and all the targets categorized into this Pfam term. Afterwards we generated a heat map according to the matrix of the values between 573 miRNAs and 73 Pfam terms. As shown in Fig. 5, some miRNAs, especially those of the same family show functional similarity by targeting proteins of the same family. We made a zoom-in view of 4 members of mir-10 family, 5 of mir-17 and 4 of mir-34 as examples to elucidate the functional relevance. Some obvious preference between miRNA families and protein families are also shown in Table 4. It can be seen some members of mir-17 family specifically target proteins related to cyclin-dependent kinase like those from “PF02234”, and members of mir-10 family tend to target some receptors like proteins from “PF01030” and “PF00757” which are involved in signaling transduction.

### The miR-21 and miR-519d-regulated CePPIN

**Figure 6:**
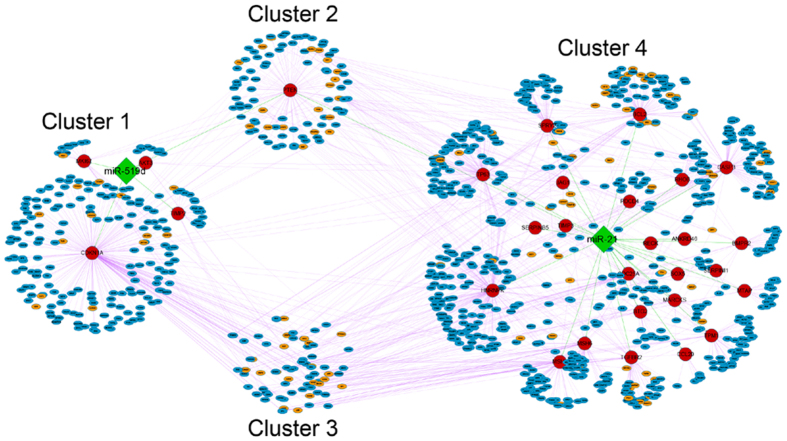
The visualization of miR-519d and miR-21-regulated CePPIN. The green diamond nodes are two miRNAs, namely miR-519d and miR-21. The red circle nodes are direct targets of miR-519d and miR-21 respectively. The eclipse nodes in small size are co-expressed proteins of those targets of miR-519d and miR-21. Among these eclipse nodes, the orange nodes are targets of other miRNAs except miR-519d and miR-21, and the blue nodes are non-targets. MiRNA-target regulation is denoted as green edges and PPIs are denoted as purple edges. The meaning of the four clusters is discussed in Results.

To concretely explain the similarities and differences between miRNAs specifically regulating only one cancer and miRNAs generally regulating various cancers in our work, we extracted miR-519d and miR-21-regulated CePPIN for further analysis. MiR-519d specifically regulates hepatocellular carcinoma and it is one of the miRNAs which have most targets among miRNAs regulating only one cancer. MiR-21 regulates 12 cancers including hepatocellular carcinoma in our data. The sub-network that miR-519d and miR-21 jointly regulates is visualized in Fig. 6. We found that miR-519d and miR-21 share the same target PTEN and they also have their own exclusive targets. Moreover, the specific targets of miR-519d and miR-21 also have common and exclusive co-expressed proteins respectively. In that case, we divided these nodes into 4 clusters. Cluster 1 denotes miR-519d-regulated exclusive targets and the exclusive co-expressed proteins of these targets. Cluster 2 denotes PTEN and its co-expressed proteins. Cluster 3 denotes the shared co-expressed proteins of exclusive targets for miR-519d and miR-21 respectively. Cluster 4 denotes miR-21-regulated exclusive targets and the exclusive co-expressed proteins of these targets. Figure 6 also demonstrates the crosstalk motifs between the two miRNAs in CePPIN. That is, some of miR-519d targets are the co-expressed proteins of miR-21 and other miRNA targets. Some of miR-21 targets are also the co-expressed proteins of miR-519d and other miRNA targets.

## Discussion

miRNAs function as post-transcriptional regulators by specifically inducing genesilencing in human cells. Their targets mostly are important nodes in human PPIN and abnormal miRNA regulation will make a dynamic change in PPIN, thus to activate or inhibit some signaling pathways related to carcinogenesis.

In our work, we elucidated the miRNA regulation roles in PPIN based on topological and functional analysis. We integrated miRNA target data with PPI data to construct miRNA-regulated CePPIN related to 17 cancers. In CePPIN, the node with the highest degree and betweenness centrality is APP protein which has been a promising research hotspot recently for its close relationship with Alzheimer disease (AD). But in our dataset, only one miRNA, namely miR-20a, targets APP to induce ovarian cancer. In fact, APP protein has been reported to play an important role in other kinds of human cancers^25^,^26^,^27^,^28^,^29^. It has been proved that the N-terminal domain of APP can exhibit growth factor-like function^30^, which to a certain extent explains how APP regulates proliferation and migration of cancer cells. Nevertheless, the precise mechanism of APP on tumor cells still remains to be disclosed.

In network topology, degree and betweenness centrality are two top-priorities to evaluate the importance of a node. Usually, nodes with high degrees are called hubs and with high betweenness centralities are called bottlenecks. But the exact measurement of hubs and bottlenecks may still remain ambiguous to the best of our knowledge. In scale-free networks, we usually define nodes with degrees much higher than average as hubs^31^. In Wang’s work^19^, they defined the top 10% of proteins with the highest degree as hubs. Using his method, 978 proteins in our CePPIN are denoted as hubs with all their degrees of higher than 10 and also much higher than the average degree of about 7.5 in CePPIN, so 403 of these 978 hubs are miRNA targets. Meanwhile, betweenness centrality might be a more significant indicator of essentiality of a node than degree^32^ and the betweenness centrality of a node is correlated to its degree in network. We found that most of the miRNA targets have highest degrees and betweenness centralities in CePPIN. They usually act as hub and bottleneck regulators. Further we found the four topological parameters of a target in CePPIN, including degree, betweenness centrality, clustering coefficient and closeness centrality are closely related to the number of miRNAs regulating this target. Targets regulated by more miRNAs obviously tend to have higher degrees, betweenness centralities and closeness centralities. Owing to the existence of these miRNA targets, the CePPIN remains robust against random attack, but it is more sensitive to even slight changes of miRNA expression and more efficient to transfer the changes to other proteins, which can make a global influence on the whole PPIN. Moreover, clustering coefficients of these targets are much lower than the average value, although it shows no significance from the result of rank sum test. It may indicate that targets regulated by more miRNAs are more likely to be intermodular hubs than intramodular hubs. Intermodular hubs are involved in a wider variety of cellular processes and are more efficient in mediating the transmission of perturbation and are more important in network cooperation for PPIN^10^,^33^. Based on the above-mentioned, miRNA targets are mostly the key regulators in human PPIN, which can to some extent demonstrate that miRNA regulation plays a highly important role in human PPIN.

For functional analysis, we focused on the biological process, molecular function and KEGG pathway information for the miRNA targets and expected to find the clear relationships among the 3 kinds of functional information. The results show that most enriched molecular function terms are about transcription factor activity, kinase activity, growth factor binding and SMAD binding (shown in Supplementary Table S2), together with some about DNA binding transcription regulator activity and so on. Correspondingly, the most enriched biological process terms are about regulation of transcription, kinase activity, phosphorylation and cell surface receptor linked signal transduction. Besides, some terms related to cell cycle like “GO: 0042127~regulation of cell proliferation” and “GO: 0043067~regulation of programmed cell death” are also among the most enriched. Moreover, terms about cell adhesion, migration and angiogenesis (shown in Supplementary Table S2) are related tightly to tumor metastasis and progression. Using KEGG pathway analysis, many cancer-related pathways were found like MAPK, VEGF, TGFβ signaling pathways which are induced by kinases and growth factors. Besides, some cell cycle, cellular immune response and tumor metastasis related pathways are also among the most enriched. All of these indicate that most miRNA targets possess diverse molecular functions such as activities of transcription factor, kinase, growth factor, ubiquitin and methylase. Therefore these targets are involved in various cellular processes including proliferation, apoptosis, localization and migration which can be the major causes of tumorigenesis and cancer progression. We further found that targets regulated by different numbers of miRNAs may have differences in their functions, but similarities among these targets are also revealed (Supplementary Table S3). Moreover, the targets regulated by more miRNAs show a higher functional similarity and maybe a weaker functional diversity, especially viewed from the results of GO: mf and KEGG pathway analysis (Fig. 4B).

For a supplement of functional analysis, we performed family analysis between miRNAs and targets according to Pfam classification. The analysis results show that miRNAs belonging to the same family also have a preference for the targets from the same protein family (Table 4). In Fig. 5, we made a zoom-in view of 4 members of mir-10 family, 5 members of mir-17 family and 4 members of mir-34 family as an example. As is shown, mir-10 family prefers to target proteins of “PF00757: Furin-like” and “PF01030: Recep_L_domain” families, mir-17 family prefers to target proteins of “PF02234: Cyclin-dependent kinase inhibitor” and mir-34 family prefers to target proteins of “PF01056: Myc_N” and “PF00452: Bcl-2” family. As is known, miRNAs of the same family share conserved seed regions in their 5′ ends that play important role in specifically binding to mRNA targets, so miRNAs of the same family possibly share targets of the same protein family or in the same pathway^5^,^34^. Our work made a validation of the functional coordination of these miRNAs. Meanwhile, targets containing domains like Bcl, Myc, cyclin, protein kinase, P53 *et al*. are more likely to be regulated by miRNAs, most of which relate closely to cell cycle process, thus to induce or inhibit cancers.

Finally, we extracted miR-519d and miR-21-regulated CePPINs as an example to further testify our analysis and findings. MiR-519d only regulates hepatocellular carcinoma and miR-21 is involved in 12 cancers including hepatocellular carcinoma. It can be found that miR-519d also have targets with high degrees and betweenness centralities such as CDKN1A (degree = 253, betweenness centrality = 0.3611) and shared one target PTEN (degree = 92, betweenness centrality = 0.1124) with miR-21. These proteins are known to have a close relationship with various kinds of cancer. Interestingly, some co-expressed proteins are subunits or activators of proteasome such as PSMA6, PSMA7 and PSME3. The three proteins are all co-expressed proteins of TIMP2 (degree = 22, betweenness centrality = 0.0308) that is one of miR-519d targets. All of them are closely related to Hepatitis C virus (HCV) pathogenesis which is a major cause of chronic liver disease that frequently leads to hepatocellular carcinoma^35^,^36^,^37^,^38^,^39^. Furthermore, the inhibition of ubiquitin–proteasome system has an anti-cancer effect by a restoration of cell cycle arrest and/or apoptotic cell death, which has already been used in cancer therapy^40^. Therefore, we can conclude that miR-519d may induce hepatocellular carcinoma through a regulation of proteasome, but the exact mechanism still need to be revealed in future researches. In addition, we found that many targets of some miRNAs could be the co-expressed proteins of targets of other miRNAs, thus to form crosstalk motifs between these miRNAs in PPIN^41^. Obviously, it will increase miRNA synergy and complexity of miRNA regulation in PPIN.

## Conclusion

In conclusion, we constructed miRNA-regulated CePPIN of 17 cancers and elucidated the roles that miRNA regulation plays in PPIN based on topological and functional analysis on CePPIN. MiRNA targets are mostly key regulators with high degrees, betweenness centralities, closeness centralities and low clustering coefficients. These targets are involved in various cellular processes related to cancer and other kinds of human diseases. MiRNAs belonging to the same family also have a preference for targets from the same protein family and a synergy effect does exist between miRNAs in regulating PPIN.

## Material and Methods

### Dataset of miRNA and targets

Dataset of miRNAs and corresponding targets were downloaded from oncomiRDB (v-1.1-20131217). This database collects manually curated 2259 entries of cancer-related miRNA regulations from literatures and covers more than 300 experimentally verified miRNAs and 829 targets across 25 cancer tissues^42^.Totally, we removed cancer types with insufficient miRNA and target information and then selected 17 representative cancers with 288 miRNAs and 573 targets.

### Dataset of PPIN

In our study, the construction of PPIN was generated by Network Analyst^43^ which contains the initial human PPI data from InnateDB^22^. We mapped 573 targets of 288 miRNAs to the PPI data to find the co-expressed proteins of these targets. 552 targets and their co-expressed proteins were extracted from the original data, since 21 targets were discarded for lack of interactome information. Then, we used selected PPIs to construct the miRNA-regulated CePPIN for 17 cancers.

### Network visualization

Network Analyst and Cytoscape (version 3.2.0)^44^ were used for network visualization and sub-network extraction. Moreover, network analyzer in Cytoscape was used to compute the topological parameters of CePPIN.

### Topological parameters of PPIN

Over the years, some computational concepts from network topology have been used to quantitatively describe the characterization of various biological networks. Especially for PPIN, this method makes it more effective to identify important molecules, analyze functional modules or pathways and uncover the mechanism of molecular interaction^45^, even to predict new molecular interaction^46^,^47^.

In our work, we used four parameters to describe the characterization of the nodes in CePPIN, namely degree, betweenness centrality, clustering coefficient and closeness centrality respectively. The formulas and descriptions of the four parameters are displayed in Table 5. Degree of a node in network is characterized by the number of its adjacent nodes. In scale-free network, we usually define the nodes with degrees much higher than the average degree of the whole network as hubs^31^. Betweenness centrality measures the efficiency of a node in information spreading and nodes with high betweenness centralities are called bottlenecks. Mostly, nodes with high degrees tend to have high betweenness centralities and the two parameters are considered to be the best predictor of the essentiality of a node for network robustness, cooperation and communication. Clustering coefficient measures the clustering tendency of a node with its adjacent nodes in network. Closeness centrality can be calculated as the reciprocal of the average shortest path length between a node and any other node in network. Nodes with higher closeness centralities are closer to other nodes in location and can also be more efficient to transmit information.

### Functional analysis

We used The Database for Annotation, Visualization and Integrated Discovery (DAVID) v6.7^48^ and Pfam^49^ to get functional annotation and family classification information for the 573 miRNA targets. After removing redundant family information, we totally got 73 Pfam terms to categorize the 287 of the 573 targets. Then, we calculated the ratio of the number of targets regulated by each miRNA and the total number of targets categorized into a Pfam terms, thus to generate a 73*288 matrix. miRNA family data was downloaded from miRBase (release v21)^50^.

## Additional Information

**How to cite this article**: Liu, Z. *et al*. Dissecting the regulation rules of cancer-related miRNAs based on network analysis. *Sci. Rep.* **6**, 34172; doi: 10.1038/srep34172 (2016).

## Supplementary Material

Supplementary Information

## Figures and Tables

**Table 1:** Top10 proteins by degree in CePPIN.

Symbol	Name	Degree	Betweenness centrality
APP	Amyloid precursor protein	1960	0.2206
HNF4A	Hepatocyte nuclear factor 4, alpha	1761	0.1858
ELAVL1	ELAV like RNA binding protein 1	1732	0.1931
ESR1	Estrogen receptor 1	799	0.0434
MYC	v-myc avian myelocytomatosis viral oncogene homolog	714	0.0441
FN1	Fibronectin 1	689	0.0323
TP53	Tumor protein p53	661	0.0446
SIRT7	Sirtuin 7	655	0.0350
CAND1	Cullin-associated and neddylation-dissociated 1	619	0.0269
YWHAZ	Tyrosine 3-monooxygenase/tryptophan 5-monooxygenase activation protein, zeta	616	0.0329

**Table 2:** Topological parameters of all nodes and miRNA targets only in CePPIN.

	Degree	Betweenness centrality	Clustering coefficient	Closeness centrality
All	7.5060 ± 43.0995	2.21 × 10^−4^ ± 3.82 × 10^−3^	0.1663 ± 0.2761	0.3197 ± 0.0322
miRNA targets	72.2989 ± 166.4770	0.0036 ± 0.0156	0.0855 ± 0.1170	0.3572 ± 0.0362

**Table 3:** Topological paremeters of miRNA targets in the three groups.

	Degree	Betweenness centrality	Clustering coefficient	Closeness centrality
Group 1	59.8652 ± 160.4425	0.0028 ± 0.0159	0.0908 ± 0.1317	0.3530 ± 0.0343
Group 2	86.0130 ± 176.7134	0.0042 ± 0.0162	0.0752 ± 0.0873	0.3608 ± 0.0391
Group 3	127.4048 ± 162.8453	0.0050 ± 0.0095	0.0785 ± 0.0649	0.3799 ± 0.0298

**Table 4:** Preference of miRNA families for target families.

miRNA family	miRNA	protein family
mir-17 family	miR-17, miR-20a, miR-93, miR-106b, miR-17-5p	PF02234
mir-146 family	miR-146a, miR-146b-5p	PF01030, PF00757
mir-10 family	miR-99a, miR-125a, miR-125b, miR-125a-5p	
mir-148 family	miR-148a, miR-152	
mir-199 family	miR-199a, miR-199b-5p	
mir-133 family	miR-133a, miR-133b	
mir-15 family	miR-15, miR-15a, miR-15b, miR-16, miR-195	PF02984, PF00134
mir-181 family	miR-181a, miR-181b	PF00454
	miR-181a, miR-181d	PF00452, PF02180
mir-29 family	miR-29b, miR-29c, miR-29a	PF01056
	miR-29b, miR-29, miR-29c	PF00452
mir-200 family	miR-200, miR-200a, miR-200b	PF00046
mir-10 family	miR-100, miR-99b, miR-99a	PF00454
mir-34 family	miR-34a, miR-34c, miR-34c-5p	PF01056
	miR-34a, miR-34c-5p, miR-34	PF00452

**Table 5:** Functions and descriptions of the four topological parameters.

Parameter	Function	Description
Degree	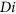	the number of links to node *i*.
Betweenness centrality	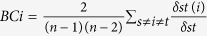	*s* and *t* are nodes in network different from *i*. 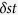 denotes the number of shortest path from *s* to *t*, and 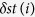 is the number of shortest paths from *s* to *t* that *i* lies on. *n* is the total number of nodes in network. The betweenness centrality of a node is a number between 0 and 1.
Clustering coefficient	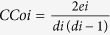	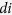 is the number of neighbors of *i*, and 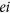 is the number of connected pairs between all nodes of *i*.
Closeness centrality	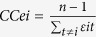	*n* is the total number of nodes in network. *t* is the node different from *i*, and 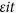 is the shortest path length between *i* and *t*.
