# Supplementary material for: Dissecting the regulation rules of cancer-related miRNAs based on network analysis
Source: Sci Rep. 2016 Oct 3;6:34172. doi: 10.1038/srep34172 (PMC5046108; doi:10.1038/srep34172)
Supplement: Supplementary Information [file srep34172-s1.pdf]

Dissecting the regulation rules of cancer-related miRNA based on network analysis

Zhongyu Liu[1], Yanzhi Guo[2]*, Xuemei Pu[2], Menglong Li[2]*

[1]College of Life Science, Sichuan University, Chengdu, Sichuan 610064, PR China

[2]College of Chemistry, Sichuan University, Chengdu, Sichuan 610064, PR China

Corresponding authors marked with *

Yanzhi Guo

Menglong Li

Tel: +86 28 85413330

Fax: +86 28 85412356

E-mail address:yzguo@scu.edu.cn

liml@scu.edu.cn

Supplementary Tables:

Supplementary table S1 | Topological parameters of top 500 proteins in CePPIN

Name	Target	Degree	BC	CCo	CCe	ASP
APP	Y	1960	0.2206	0.0009	0.4502	2.2214
HNF4A	Y	1761	0.1858	0.0009	0.4376	2.2855
ELAVL1	Y	1732	0.1931	0.0018	0.4586	2.1808
ESR1	Y	799	0.0434	0.0117	0.4438	2.2532
MYC	Y	714	0.0441	0.0099	0.4317	2.3163
FN1	Y	689	0.0323	0.0088	0.4247	2.3547
TP53	Y	661	0.0446	0.0166	0.4604	2.1719
SIRT7	Y	655	0.035	0.0035	0.4179	2.3927
CAND1	Y	619	0.0269	0.0077	0.3993	2.5043
YWHAZ	Y	616	0.0329	0.012	0.4248	2.354
CDK2	Y	569	0.0253	0.0134	0.4269	2.3423
HDAC1	Y	541	0.0306	0.0184	0.4309	2.3206
SP1	Y	540	0.0387	0.0157	0.4361	2.2929
VCP	Y	440	0.0226	0.011	0.4133	2.4193
UBC	N	413	0.0485	0.0315	0.4899	2.0414
CUL2	Y	406	0.0182	0.0115	0.4126	2.4234
BRCA1	Y	404	0.0223	0.0221	0.4125	2.4244
VHL	Y	398	0.0213	0.0118	0.4269	2.3424
EGFR	Y	391	0.0185	0.0183	0.3966	2.5214
SRC	Y	338	0.0135	0.0226	0.392	2.5509
CTNNB1	Y	330	0.0211	0.0306	0.4603	2.1723
SMAD3	Y	330	0.0176	0.0274	0.44	2.273
CSNK2A1	Y	323	0.018	0.0168	0.4453	2.2455
SIRT1	Y	314	0.0171	0.0208	0.4277	2.3381
SMAD2	Y	313	0.0136	0.0186	0.4048	2.4705
JUN	Y	279	0.0148	0.0397	0.4451	2.2468
ABL1	Y	276	0.0152	0.0203	0.4038	2.4763
AR	Y	276	0.0124	0.0277	0.4086	2.4476
CRK	Y	257	0.0092	0.0179	0.3634	2.752
CDKN1A	Y	249	0.0133	0.0327	0.4451	2.2468
AKT1	Y	248	0.0094	0.0309	0.4115	2.43
CREB1	Y	245	0.0159	0.0178	0.4076	2.4535
TRAF2	Y	241	0.0109	0.0075	0.3556	2.8123
NFKB1	Y	240	0.0084	0.0242	0.3779	2.6463
PIK3R1	Y	235	0.0095	0.0286	0.3836	2.6071
RB1	Y	224	0.0057	0.0412	0.3892	2.5694
STAT1	Y	223	0.0102	0.0287	0.411	2.433
STAT3	Y	222	0.0088	0.0377	0.4083	2.4493
FOS	Y	214	0.0097	0.0346	0.4128	2.4225
SRPK1	Y	213	0.013	0.0139	0.4064	2.4605

VIM	Y	206	0.0099	0.0181	0.403	2.4811
HNRNPK	Y	189	0.0049	0.0414	0.4083	2.4493
HDAC4	Y	188	0.0111	0.0328	0.4395	2.2753
MAPK1	Y	187	0.0065	0.0347	0.4047	2.4707
NOTCH1	Y	181	0.0068	0.02	0.3928	2.5456
PXN	Y	181	0.0047	0.0223	0.367	2.7248
HDGF	Y	178	0.0029	0.0062	0.3526	2.8363
SMAD4	Y	170	0.0074	0.0424	0.4201	2.3801
IKBKB	Y	169	0.0035	0.039	0.3725	2.6844
PLK1	Y	168	0.0087	0.0183	0.3952	2.5302
PLCG1	Y	165	0.004	0.0216	0.3559	2.81
E2F1	Y	161	0.0037	0.0544	0.3797	2.6334
PPP2CA	Y	159	0.0067	0.0289	0.3837	2.6061
ERBB2	Y	157	0.005	0.0479	0.3962	2.5239
HIF1A	Y	157	0.0054	0.0586	0.4138	2.4165
CEBPB	Y	155	0.0071	0.0503	0.4209	2.3759
YBX1	Y	154	0.0043	0.0399	0.4009	2.4946
EZH2	Y	149	0.003	0.0465	0.3794	2.6358
CDKN2A	Y	148	0.0034	0.0419	0.3812	2.6232
HGS	Y	148	0.0057	0.012	0.3781	2.6451
RAC1	Y	148	0.0054	0.0133	0.3592	2.7843
YY1	Y	147	0.0058	0.0412	0.4162	2.4024
ILK	Y	146	0.0037	0.0109	0.3596	2.7808
MAP3K1	Y	146	0.0022	0.0272	0.3663	2.7303
CDC42	Y	144	0.0054	0.0152	0.3759	2.6603
NFKBIA	Y	142	0.0025	0.0395	0.3737	2.6761
MLH1	Y	140	0.0065	0.0166	0.3766	2.6557
APC	Y	136	0.0046	0.0118	0.3609	2.7705
ETS1	Y	133	0.0065	0.0334	0.4097	2.4405
RAF1	Y	133	0.0039	0.041	0.3728	2.6823
SRSF1	Y	131	0.0047	0.0571	0.4323	2.3132
CDH1	Y	130	0.0028	0.0304	0.3648	2.7412
CDK4	Y	130	0.0033	0.0425	0.3909	2.5582
TP63	Y	129	0.0035	0.0203	0.3624	2.7591
MAPK3	Y	127	0.0035	0.0292	0.3898	2.5656
GJA1	Y	126	0.0029	0.0156	0.3611	2.7689
VDR	Y	124	0.0035	0.0514	0.3948	2.533
PPARG	Y	123	0.0046	0.0399	0.3881	2.5769
ATM	Y	117	0.0031	0.0455	0.3926	2.547
MAP3K7	Y	117	0.0049	0.0224	0.3725	2.6849
CCND1	Y	116	0.0022	0.0871	0.4012	2.4926
PTK2	Y	116	0.0055	0.0708	0.4267	2.3436
BCL2	Y	114	0.0025	0.0483	0.3674	2.7217
MYH9	Y	114	0.0034	0.037	0.3955	2.5286

PRKCA	Y	113	0.0047	0.0267	0.3842	2.6027
CDK6	Y	109	0.0033	0.0423	0.3833	2.6088
NCOA3	Y	108	0.0029	0.0827	0.4152	2.4083
RHOA	Y	108	0.0024	0.0116	0.3475	2.878
TLE1	Y	108	0.0038	0.0216	0.3602	2.776
EZR	Y	107	0.0049	0.0332	0.3928	2.5455
EP300	N	103	0.0023	0.1411	0.4089	2.4457
PTP4A3	Y	102	0.0031	0.0076	0.3627	2.7568
ESR2	Y	101	0.0011	0.0448	0.3636	2.7506
KDM1A	Y	100	0.0012	0.0586	0.3706	2.6984
CASP3	Y	99	0.0041	0.0418	0.4052	2.4677
MTOR	Y	99	0.0027	0.046	0.377	2.6524
NCOR2	Y	99	0.0019	0.0932	0.3896	2.5668
CCNB1	Y	98	0.0021	0.0614	0.3783	2.6437
HSP90AA1	N	97	0.0037	0.0868	0.4281	2.3361
MSH2	Y	96	0.0014	0.0612	0.3766	2.6552
DNMT1	Y	94	0.0012	0.0917	0.3781	2.6449
MED1	Y	93	0.0025	0.0697	0.4028	2.4828
IQGAP1	Y	92	0.0019	0.0628	0.3821	2.6171
IRAK1	Y	92	0.0018	0.0306	0.3559	2.8098
JAK2	Y	92	0.0017	0.075	0.3611	2.7695
PIK3R2	Y	92	0.0021	0.0839	0.3863	2.5886
PTEN	Y	92	0.0037	0.0368	0.3837	2.6064
BCL2L1	Y	91	0.0035	0.0508	0.3886	2.5736
PHB	Y	91	0.0032	0.0518	0.3909	2.5579
ITGB1	Y	88	0.0036	0.0374	0.3775	2.6487
SMAD7	Y	88	0.0017	0.0721	0.3642	2.7459
CDKN1B	Y	87	0.0019	0.0922	0.4114	2.4309
YAP1	Y	86	0.0027	0.0451	0.3805	2.6282
CTBP1	Y	85	0.002	0.0667	0.3835	2.6077
PAK1	Y	84	0.0032	0.0522	0.3887	2.5727
TFAP2A	Y	82	0.003	0.0623	0.411	2.433
EGR1	N	81	0.0018	0.0966	0.3893	2.569
HMGA1	Y	81	0.0009	0.0546	0.3675	2.721
SMARCA5	Y	80	0.0014	0.0604	0.3873	2.5823
SPP1	Y	80	0.003	0.0028	0.34	2.9413
CCNE1	Y	78	0.0008	0.0839	0.3784	2.6426
FBXW7	Y	78	0.0013	0.0336	0.3523	2.8385
CCNA2	Y	77	0.0005	0.1077	0.3718	2.6897
SEC23A	Y	76	0.003	0.0319	0.3811	2.6242
DNMT3B	Y	75	0.0013	0.0818	0.3662	2.7309
SMARCD1	Y	75	0.0017	0.0404	0.3585	2.7893
SRSF2	Y	75	0.0016	0.0746	0.3886	2.5733
CLU	Y	74	0.0029	0.0255	0.3762	2.6582

FOXO3	Y	74	0.0014	0.107	0.3967	2.521
KEAP1	Y	74	0.0021	0.0141	0.3468	2.8837
SUMO2	N	74	0.0016	0.0955	0.407	2.4567
ERBB3	Y	72	0.0014	0.1002	0.3565	2.805
SUMO1	N	72	0.0023	0.1307	0.4314	2.3179
SUZ12	Y	72	0.0007	0.1045	0.3799	2.6326
HDAC2	N	71	0.0013	0.1658	0.4144	2.4131
ARHGDIA	Y	69	0.0016	0.0426	0.3614	2.7673
CREBBP	N	69	0.0017	0.1897	0.4276	2.3384
HSP90AB1	N	69	0.0018	0.098	0.3983	2.5104
RBL2	Y	69	0.0023	0.0921	0.398	2.5124
SOCS3	Y	69	0.0028	0.0533	0.3959	2.5261
RELA	N	67	0.0014	0.1452	0.3908	2.5591
SH3GL2	Y	66	0.0031	0.0121	0.365	2.7395
TGFBR1	Y	66	0.0014	0.0895	0.3811	2.6241
CDH2	Y	65	0.0016	0.0135	0.347	2.8818
GRB2	N	65	0.0022	0.1337	0.4149	2.4102
BMI1	Y	64	0.0018	0.0585	0.3897	2.5664
RAB5A	Y	64	0.0023	0.0149	0.3617	2.7651
SOCS1	Y	64	0.0013	0.0779	0.3636	2.7505
DAB2	Y	63	0.0014	0.0497	0.3802	2.6304
GNAI2	Y	63	0.0031	0.0061	0.3437	2.9094
SOX2	Y	63	0.0011	0.0492	0.3456	2.8939
APOE	Y	62	0.0037	0.0296	0.3967	2.521
EED	Y	62	0.0013	0.0788	0.37	2.7027
PTPN1	Y	62	0.0009	0.0788	0.365	2.7395
SRF	Y	62	0.0005	0.0471	0.351	2.8487
CDC25A	Y	61	0.0017	0.0754	0.4043	2.4732
MSH6	Y	61	0.0005	0.0962	0.3679	2.7178
NR0B2	Y	60	0.001	0.0847	0.3895	2.5675
RAP1A	Y	60	0.0022	0.0305	0.3783	2.6435
LAMTOR5	N	59	0.0019	0.0152	0.3632	2.7532
RASA1	Y	59	0.0015	0.083	0.3933	2.5427
CXCR4	Y	58	0.0008	0.0448	0.3584	2.7901
IGF1R	Y	58	0.0017	0.127	0.3892	2.5693
PRDX2	Y	58	0.0024	0.0526	0.3863	2.5884
TGFBR2	Y	58	0.0013	0.0901	0.3826	2.6137
HDAC3	N	57	0.0011	0.1848	0.407	2.4569
MTA1	Y	57	0.001	0.1003	0.3773	2.6508
RUNX2	Y	57	0.001	0.1103	0.3644	2.7444
TPM1	Y	57	0.0018	0.0326	0.3769	2.6532
CD44	Y	56	0.0012	0.0857	0.3837	2.6065
IL8	Y	56	0.0015	0.0857	0.3695	2.7064
IRS1	Y	56	0.0008	0.1584	0.3852	2.5961

RPS6KB1	Y	56	0.0009	0.0734	0.3927	2.5464
YES1	Y	56	0.0008	0.0578	0.3793	2.6365
CUL3	N	55	0.0014	0.0747	0.4148	2.4108
MCM5	Y	55	0.001	0.0505	0.3827	2.6132
VASP	Y	55	0.0014	0.0195	0.3487	2.8674
CASP7	Y	54	0.001	0.0412	0.3563	2.8066
COPS5	N	54	0.0021	0.1495	0.4408	2.2684
PCMT1	Y	54	0.0011	0.0321	0.3779	2.6465
PIK3CA	Y	54	0.0012	0.0783	0.3615	2.7665
AKT2	Y	53	0.0016	0.037	0.3761	2.6588
CFLAR	Y	53	0.0008	0.0537	0.3599	2.7789
SRSF9	Y	53	0.001	0.0842	0.3925	2.548
TERT	Y	53	0.0008	0.1277	0.3749	2.6673
UHRF2	Y	53	0.0005	0.111	0.3625	2.7586
CDK19	Y	52	0.0004	0.0249	0.33	3.0303
MDM2	N	52	0.0012	0.1456	0.4189	2.3871
PPARA	Y	52	0.0018	0.0483	0.3615	2.7666
PPP2R2A	Y	52	0.0009	0.0483	0.3696	2.7057
RPS6KA5	Y	52	0.0018	0.0528	0.3991	2.5057
ARNT	Y	51	0.0013	0.0722	0.3783	2.6435
FSCN1	Y	51	0.0007	0.0384	0.3621	2.7618
GABPA	Y	51	0.0023	0.0612	0.3986	2.5089
LARP1	Y	51	0.0008	0.0855	0.3795	2.6348
MET	Y	51	0.0012	0.0714	0.3559	2.8097
PPARGC1A	Y	51	0.0004	0.1114	0.3791	2.6381
PTMA	Y	51	0.0011	0.051	0.3723	2.6859
TCF4	Y	51	0.0009	0.0588	0.3498	2.8591
TGFB1	Y	51	0.0029	0.0792	0.3993	2.5041
ATF2	N	50	0.001	0.1682	0.4181	2.3916
GSK3B	N	50	0.0017	0.1453	0.4369	2.2888
ING1	Y	50	0.0006	0.1102	0.3808	2.6259
SHMT2	Y	50	0.0011	0.0482	0.3759	2.6604
SPRY2	Y	50	0.0026	0.0343	0.3717	2.6903
YWHAQ	N	50	0.0008	0.1461	0.4028	2.4824
AHR	Y	49	0.0009	0.0935	0.3627	2.7574
IGF2BP1	Y	49	0.0006	0.0604	0.3648	2.7413
KIT	Y	49	0.0006	0.0918	0.3553	2.8143
PRKCE	Y	49	0.0013	0.0723	0.3915	2.5545
FOSL1	Y	48	0.0004	0.0984	0.3593	2.7829
MAPRE1	Y	48	0.0011	0.047	0.3771	2.6518
MDM4	Y	48	0.0007	0.125	0.3836	2.6068
PRKACB	Y	48	0.001	0.0115	0.3434	2.9122
SKI	Y	48	0.0008	0.1516	0.3865	2.5875
KIAA0101	N	47	0.0005	0.1018	0.3888	2.5717

NME1	Y	46	0.0015	0.0203	0.37	2.7025
BCL3	Y	45	0.0003	0.1404	0.3611	2.7689
BRAF	Y	45	0.0003	0.1	0.3515	2.845
MECP2	Y	45	0.0009	0.0747	0.372	2.6881
PPP3CA	Y	45	0.0011	0.0162	0.3451	2.8981
UBE2I	N	45	0.0008	0.1737	0.3962	2.5243
USF2	Y	45	0.0018	0.0343	0.3864	2.5878
BIRC5	Y	44	0.0005	0.0793	0.3749	2.6673
CUL1	N	44	0.0005	0.1416	0.3933	2.5427
ESRRA	Y	44	0.0007	0.0391	0.351	2.8491
GIT2	Y	44	0.0009	0.0106	0.3351	2.9842
CCND3	Y	43	0.0009	0.0831	0.3791	2.6379
FTH1	Y	43	0.0009	0.0066	0.3402	2.9396
HES1	Y	43	0.001	0.0698	0.3714	2.6923
HSPA4	N	43	0.001	0.1528	0.4109	2.4334
MCL1	Y	43	0.0002	0.0975	0.3498	2.8588
PIK3R3	Y	43	0.001	0.0875	0.3648	2.7414
SMARCA4	N	43	0.0006	0.2071	0.3901	2.5631
CYLD	Y	42	0.0014	0.0441	0.3641	2.7463
DNMT3A	Y	42	0.0006	0.1161	0.3537	2.8272
FOXO1	Y	42	0.0006	0.1324	0.3854	2.5947
HSPA8	N	42	0.001	0.1905	0.4139	2.4159
PIN1	N	42	0.0009	0.1905	0.4175	2.3952
ATG7	Y	41	0.0012	0.0695	0.3671	2.7244
HSPA1B	N	41	0.0009	0.1488	0.4085	2.4482
BCL2L11	Y	40	0.001	0.0769	0.3719	2.689
LEF1	Y	40	0.0004	0.1359	0.3568	2.8029
MITF	Y	40	0.0021	0.0564	0.3927	2.5462
PGRMC1	Y	40	0.0007	0.0077	0.3421	2.9235
PML	N	40	0.0003	0.2295	0.3817	2.6201
BAG3	Y	39	0.0007	0.0243	0.3415	2.9279
FGFR1	Y	39	0.0012	0.0864	0.3504	2.8539
MAPK8	N	39	0.0008	0.1565	0.3977	2.5142
MIF	Y	39	0.0005	0.0499	0.3591	2.785
PIM1	Y	39	0.0006	0.0918	0.3932	2.5431
PTGS2	Y	39	0.0004	0.1363	0.3776	2.6482
VEGFA	Y	39	0.0011	0.1484	0.3815	2.6209
YWHAG	N	39	0.0008	0.1552	0.4106	2.4354
COPS8	Y	38	0.0004	0.0797	0.3698	2.7044
GAB2	Y	38	0.0002	0.1607	0.3482	2.8723
HIST1H4E	N	38	0.0009	0.1821	0.4198	2.382
IKBKG	N	38	0.0005	0.1209	0.3739	2.6748
MAP3K11	Y	38	0.0009	0.0782	0.3704	2.6999
TAGLN2	Y	38	0.0013	0.0583	0.3977	2.5146

TRAF6	N	38	0.0012	0.0868	0.4066	2.4594
ACTB	N	37	0.001	0.2057	0.4226	2.3662
GSK3A	Y	37	0.0012	0.0691	0.3754	2.6637
IRS2	Y	37	0.0004	0.1922	0.3853	2.5956
THBS1	Y	37	0.001	0.0586	0.3684	2.7143
YWHAE	N	37	0.0003	0.1471	0.3767	2.6545
COL1A1	Y	36	0.0014	0.0984	0.3787	2.6403
FGFR3	Y	36	0.0013	0.0016	0.331	3.0208
GSTP1	Y	36	0.0004	0.0667	0.3633	2.7523
HSPA5	N	36	0.0008	0.2	0.4052	2.4679
PLAUR	Y	36	0.0007	0.0206	0.3542	2.823
RABEP1	Y	36	0.0007	0.027	0.3416	2.9273
STUB1	N	36	0.0008	0.1841	0.4013	2.4921
YWHAB	N	36	0.0009	0.1683	0.4295	2.3284
CHUK	N	35	0.0006	0.2134	0.3939	2.539
MAPK7	Y	35	0.0011	0.0387	0.3802	2.6305
MYO6	Y	35	0.0005	0.0403	0.3549	2.8175
PRKDC	N	35	0.0006	0.2437	0.4054	2.4666
RDX	Y	35	0.001	0.0723	0.3698	2.7044
RHOB	Y	35	0.0008	0.0235	0.3588	2.7873
RUNX3	Y	35	0.0004	0.1109	0.3529	2.8338
SP3	N	35	0.0003	0.1697	0.3696	2.7059
BUB1	Y	34	0.0009	0.0481	0.3538	2.8262
ITGA4	N	34	0.0002	0.1105	0.3573	2.7985
KAT2B	N	34	0.0002	0.2282	0.3723	2.6862
KRAS	Y	34	0.0017	0.0481	0.3898	2.5656
PARP1	N	34	0.0005	0.2389	0.4026	2.4841
SNX1	Y	34	0.0016	0.0784	0.3683	2.7155
CALM3	N	33	0.0005	0.1439	0.3959	2.5257
CAV1	N	33	0.001	0.1326	0.4136	2.4176
CBL	N	33	0.0003	0.2045	0.358	2.7933
CLINT1	Y	33	0.0007	0.0208	0.36	2.7781
DAPK1	Y	33	0.0003	0.0265	0.3468	2.8835
MMP2	Y	33	0.0009	0.0966	0.3602	2.7759
NEDD9	Y	33	0.0005	0.1212	0.3732	2.6795
NPM1	N	33	0.0008	0.233	0.4069	2.4573
PEBP1	Y	33	0.0006	0.0587	0.3541	2.8243
RAP1B	Y	33	0.0006	0.0341	0.3742	2.6724
SNAI1	Y	33	0.0008	0.1212	0.3529	2.8337
XRCC6	N	33	0.0004	0.1799	0.3855	2.5942
BTRC	N	32	0.0006	0.1694	0.4031	2.481
E2F3	Y	32	0.0003	0.0605	0.3629	2.7554
E2F6	Y	32	0.0006	0.0665	0.3643	2.7452
FOXM1	Y	32	0.0002	0.1754	0.3617	2.7649

KLF5	Y	32	0.0003	0.2339	0.3864	2.5879
NEDD8	N	32	0.0006	0.1734	0.4086	2.4474
SHC1	N	32	0.0004	0.2661	0.3833	2.609
CDK1	N	31	0.0003	0.2495	0.3913	2.5559
CEBPA	N	31	0.0006	0.2946	0.406	2.463
EPB41L3	Y	31	0.0011	0.0796	0.3755	2.6634
KAT5	N	31	0.0005	0.1935	0.3953	2.5297
MUC1	Y	31	0.0002	0.228	0.3659	2.7331
PCNA	N	31	0.0002	0.1849	0.3697	2.7046
PLAU	Y	31	0.0005	0.0796	0.3605	2.7735
PNP	Y	31	0.0007	0.0301	0.3552	2.8154
SERPINE1	Y	31	0.0005	0.1097	0.3694	2.707
SKP2	N	31	0.0005	0.2796	0.4088	2.446
STMN1	Y	31	0.0005	0.0753	0.3572	2.7994
ASAP1	Y	30	0.0011	0.1586	0.3677	2.72
EPHA2	Y	30	0.0017	0.1126	0.4082	2.4499
FGF2	Y	30	0.0007	0.0483	0.349	2.8651
FYN	N	30	0.0001	0.1563	0.3533	2.8304
GNB2L1	N	30	0.0005	0.1816	0.3818	2.619
ITGA5	Y	30	0.0008	0.0575	0.3483	2.8708
KLF4	Y	30	0.0005	0.1839	0.3818	2.619
PDCD4	Y	30	0.0007	0.0759	0.3867	2.5857
PRAME	Y	30	0.0001	0.0437	0.3426	2.9186
PTPN11	N	30	0.0006	0.2943	0.3951	2.5309
SIN3A	N	30	0.0001	0.2046	0.365	2.7398
ABCC1	Y	29	0.0012	0	0.3368	2.969
ACVR1B	Y	29	0.0013	0.0961	0.3637	2.7498
BAK1	Y	29	0.0003	0.0788	0.3455	2.8942
BMPR2	Y	29	0.0013	0.0419	0.3417	2.9267
CBFB	Y	29	0.001	0.0542	0.3899	2.5646
CUL4B	N	29	0.0006	0.1453	0.4005	2.497
DICER1	Y	29	0.0009	0.0099	0.3448	2.9
LATS2	Y	29	0.0004	0.0517	0.3664	2.7296
MAPK14	N	29	0.0002	0.1478	0.3652	2.7385
RBBP4	N	29	0.0005	0.2857	0.4119	2.4278
RXRA	N	29	0.0002	0.3202	0.3769	2.653
CASP8	N	28	0.0006	0.164	0.3829	2.6116
GNA13	Y	28	0.0009	0.0344	0.3597	2.7803
HDAC5	N	28	0.0002	0.2222	0.3739	2.6746
PPP1CA	N	28	0.0005	0.1958	0.3943	2.5364
RBX1	N	28	0.0005	0.1667	0.4017	2.4892
SATB1	Y	28	0.0001	0.1058	0.3486	2.8684
SMURF1	N	28	0.0004	0.2011	0.3891	2.5698
VCAM1	N	28	0.0003	0.0899	0.3729	2.6818

XPO1	N	28	0.0007	0.1799	0.4115	2.4304
AXL	Y	27	0.0006	0.1368	0.3644	2.7441
CBX7	Y	27	0.0004	0.0484	0.3293	3.0369
HBP1	Y	27	0.0009	0.0712	0.3721	2.6877
ID1	Y	27	0.0004	0.0627	0.3448	2.9003
MMP9	Y	27	0.0004	0.1197	0.3425	2.9193
MYCN	Y	27	0.0001	0.0798	0.3514	2.8458
NCK1	N	27	0.0005	0.2137	0.3706	2.698
NCOR1	N	27	0.0003	0.3077	0.3881	2.5768
NR3C1	N	27	0.0004	0.2051	0.3836	2.6069
PRKCD	N	27	0.0006	0.2279	0.4079	2.4513
RAP2A	Y	27	0.0011	0.0199	0.3585	2.7891
TUBB	N	27	0.0009	0.2279	0.4184	2.3902
TWIST1	Y	27	0.0002	0.1481	0.3529	2.834
ARHGEF2	Y	26	0.0006	0.1662	0.3801	2.6311
CEBPD	N	26	0.0001	0.2462	0.3594	2.7825
COL1A2	Y	26	0.0002	0.1077	0.3495	2.861
DDB1	N	26	0.0002	0.1908	0.3752	2.6653
EEF1A1	N	26	0.0004	0.1631	0.372	2.6882
FLNA	N	26	0.0008	0.2031	0.4005	2.4968
GAPDH	N	26	0.0004	0.1969	0.3969	2.5197
HSPD1	N	26	0.0005	0.2277	0.4057	2.4649
ING4	Y	26	0.0006	0.0615	0.3679	2.718
NEDD4	N	26	0.0002	0.1046	0.3557	2.8116
PDGFRA	Y	26	0.0003	0.1077	0.3465	2.886
PPP6C	Y	26	0.0007	0.08	0.3908	2.5587
SMAD1	N	26	0.0003	0.1908	0.3756	2.6627
SMARCC1	N	26	0.0003	0.2954	0.4007	2.4957
ARRB2	N	25	0.0002	0.14	0.3581	2.7921
CDC37	N	25	0.0004	0.14	0.3771	2.6515
CSNK2B	N	25	0.0002	0.21	0.3672	2.7236
CUX1	Y	25	0.0005	0.0333	0.34	2.9413
ESRRG	Y	25	0.0006	0.0967	0.3735	2.6774
FHL2	N	25	0.0004	0.21	0.3903	2.5621
FLT1	Y	25	0.0003	0.21	0.3556	2.8124
HSPA9	N	25	0.0006	0.2033	0.4053	2.4674
IKBKE	N	25	0.0001	0.0367	0.3414	2.9293
MKI67	Y	25	0.0003	0.0767	0.3597	2.7802
PPP1CC	N	25	0.0005	0.27	0.4087	2.4468
PRKCZ	N	25	0.0001	0.17	0.3665	2.7284
RAB14	Y	25	0.0005	0.06	0.3696	2.706
ARRB1	N	24	0.0001	0.1341	0.3577	2.7959
FLI1	Y	24	0.0003	0.0833	0.346	2.8901
GMNN	Y	24	0.0004	0.1196	0.3545	2.821

MAP3K2	Y	24	0	0	0.3368	2.9693
NCL	N	24	0.0002	0.3225	0.3984	2.51
NF1	Y	24	0.0003	0.1014	0.377	2.6522
NTRK2	Y	24	0.0004	0.0362	0.3423	2.9218
PBX2	Y	24	0.0012	0.0181	0.3412	2.9305
RAB1B	Y	24	0.0013	0.0616	0.3911	2.5569
RBL1	N	24	0.0002	0.3551	0.3877	2.5792
RUNX1	N	24	0.0002	0.3406	0.3822	2.6162
RUVBL1	N	24	0.0003	0.2174	0.3893	2.5689
SQSTM1	N	24	0.0002	0.1196	0.3602	2.7762
XBP1	Y	24	0.0004	0.0833	0.3519	2.8417
CCND2	Y	23	0	0.1779	0.3537	2.8276
CDX2	Y	23	0.0007	0.0316	0.3348	2.9866
CUL5	N	23	0.0002	0.1344	0.3747	2.6685
EGR2	Y	23	0.0009	0.0198	0.3381	2.9581
FHOD1	Y	23	0.0008	0.0632	0.3515	2.8451
HNRNPA1	N	23	0.0004	0.3162	0.4137	2.417
KPNB1	N	23	0.0006	0.2925	0.4145	2.4126
MYOD1	N	23	0.0001	0.249	0.3613	2.7678
NCOA1	N	23	0.0002	0.2964	0.3767	2.6543
NKX2-1	Y	23	0.0006	0.1146	0.3413	2.9299
NOTCH2	Y	23	0.0001	0.0395	0.3399	2.9417
NRAS	Y	23	0.0007	0.0474	0.379	2.6382
PIAS1	N	23	0.0001	0.2648	0.3701	2.7019
SET	N	23	0.0005	0.2767	0.4118	2.4284
SFN	N	23	0.0001	0.2016	0.3641	2.7464
SH3KBP1	N	23	0.0004	0.2134	0.3779	2.6462
SMARCB1	N	23	0.0004	0.253	0.3922	2.5499
TFAP2C	Y	23	0.0007	0.1739	0.3829	2.6119
TRIM28	N	23	0.0002	0.3439	0.3783	2.6436
VAV1	N	23	0.0002	0.2134	0.3532	2.8314
WASF1	Y	23	0.0003	0.0435	0.3413	2.9303
XRCC5	N	23	0.0003	0.2253	0.3897	2.5662
YWHAH	N	23	0.0002	0.1937	0.3792	2.6372
ZNF148	Y	23	0.0004	0.1897	0.3758	2.6611
BCL6	N	22	0.0004	0.1558	0.3697	2.7052
CLTC	N	22	0.0003	0.1602	0.3722	2.6866
CRKL	N	22	0.0004	0.2165	0.3863	2.5888
FOXO4	Y	22	0.0001	0.1818	0.3603	2.7756
HK2	Y	22	0.0005	0.0216	0.358	2.7932
HLA-B	N	22	0.0002	0.0606	0.3628	2.7564
HNRNPD	N	22	0.0003	0.2294	0.3821	2.6174
HNRNPU	N	22	0.0001	0.2121	0.3651	2.7388
KLF6	Y	22	0.0003	0.0909	0.369	2.7097

LASP1	Y	22	0.0003	0.0216	0.3453	2.896
LCK	N	22	0.0001	0.1645	0.3443	2.9045
MAP3K10	Y	22	0.0003	0.026	0.3315	3.0166
MARCKS	Y	22	0.0001	0.0779	0.365	2.7396
MTAP	Y	22	0.0006	0.0433	0.358	2.7932
NCOA6	N	22	0.0002	0.2987	0.3756	2.6621
PHF10	Y	22	0.0001	0.0433	0.3406	2.9362
POLR2A	N	22	0.0004	0.2121	0.4027	2.4835
ROCK1	Y	22	0.0008	0.0563	0.3638	2.7489
TERF1	N	22	0.0001	0.0476	0.3464	2.8867
TIAM1	Y	22	0.0001	0.0606	0.348	2.8737
TIMP2	Y	22	0	0.0087	0.3253	3.0738
TWF1	Y	22	0.0006	0.026	0.3605	2.7742
XIAP	N	22	0.0005	0.1299	0.39	2.5643
ZEB2	Y	22	0.0003	0.2381	0.3912	2.556
API5	Y	21	0.0009	0.019	0.3574	2.7978
ARHGEF1	Y	21	0.0003	0.0524	0.347	2.8818
BAG4	Y	21	0.0004	0.1571	0.3858	2.5921
DDX5	N	21	0.0002	0.281	0.3769	2.6529
E2F2	Y	21	0.0002	0.0571	0.3563	2.8068
GTF2I	N	21	0.0003	0.2619	0.3868	2.5851
HDAC6	N	21	0.0004	0.1571	0.3871	2.5833
HIST3H3	N	21	0.0001	0.1286	0.3427	2.9177
HNRNPM	N	21	0.0001	0.2714	0.3696	2.7054
INSR	N	21	0.0001	0.181	0.3473	2.8792
ITCH	N	21	0.0003	0.1667	0.3748	2.6682
NFYA	N	21	0.0003	0.3048	0.4015	2.4904
NKRF	Y	21	0.0001	0.0952	0.3551	2.8161
PDK1	Y	21	0.0001	0.1238	0.3548	2.8182
PPP2R1A	N	21	0.0003	0.2476	0.3877	2.5796
RUVBL2	N	21	0.0005	0.2905	0.4091	2.4445
SMARCA2	N	21	0.0001	0.2095	0.3634	2.7518
SMYD3	Y	21	0.0011	0.0905	0.3745	2.6704
TP53BP1	N	21	0.0002	0.2619	0.3683	2.7149
ZEB1	Y	21	0	0.1095	0.3401	2.94
AURKA	N	20	0.0002	0.1895	0.3776	2.6481
BARD1	N	20	0.0003	0.2842	0.3955	2.5282
BTG2	Y	20	0.0005	0.0105	0.3398	2.9425
CDK9	N	20	0.0002	0.2842	0.3813	2.6228
CFTR	N	20	0.0002	0.0421	0.3499	2.858
DDX17	N	20	0.0003	0.3895	0.4126	2.4235
H2AFX	N	20	0.0002	0.3632	0.3887	2.5728
HRAS	N	20	0.0001	0.0737	0.3518	2.8429
KAT2A	N	20	0.0001	0.3105	0.3725	2.6844

LYN	N	20	0.0001	0.2421	0.3741	2.6731
MDC1	N	20	0.0002	0.1947	0.378	2.6458
NR4A1	N	20	0.0003	0.2053	0.3573	2.7989
PAK4	Y	20	0.0002	0.1158	0.3698	2.7045
PIK3C2A	Y	20	0.0001	0.0579	0.343	2.9154
RARA	N	20	0.0001	0.1947	0.3571	2.8
ROCK2	Y	20	0.0005	0.0316	0.3596	2.7809
RPS27A	N	20	0.0006	0.1947	0.4108	2.4341
SNW1	N	20	0.0002	0.2842	0.3846	2.5998
TBP	N	20	0.0002	0.3789	0.3876	2.58
TOP1	N	20	0.0003	0.3158	0.4102	2.4378
TOP2A	N	20	0.0002	0.3105	0.3766	2.6554
TSC2	N	20	0.0001	0.1789	0.3574	2.798
UBB	N	20	0.0004	0.1632	0.393	2.5444
ACTG1	N	19	0.0002	0.1462	0.3608	2.7716
AP3B1	Y	19	0.0008	0.0234	0.3591	2.7845
APAF1	Y	19	0.0002	0.1637	0.3646	2.7427
BAX	N	19	0.0001	0.2339	0.3494	2.8624
CDKN1C	Y	19	0	0.1813	0.3443	2.9041
COL2A1	Y	19	0.001	0.0409	0.3308	3.0233

Supplementary table S2 | Functional annotation for all miRNA targets (p-value< 0.05 and FDR< 0.05)

Term	Genes	P-value
**Biological process**		
GO:0045449~regulation of transcription	200	6.22E-24
GO:0051252~regulation of RNA metabolic process	162	1.81E-25
GO:0006355~regulation of transcription, DNA-dependent	158	1.26E-24
GO:0042127~regulation of cell proliferation	146	3.18E-60
GO:0006350~transcription	143	9.32E-12
GO:0043067~regulation of programmed cell death	137	5.28E-51
GO:0010941~regulation of cell death	137	8.32E-51
GO:0042981~regulation of apoptosis	135	6.17E-50
GO:0007242~intracellular signaling cascade	132	9.15E-27
GO:0010604~positive regulation of macromolecule metabolic process	125	2.8E-39
GO:0007166~cell surface receptor linked signal transduction	124	1.37E-09
GO:0006357~regulation of transcription from RNA polymerase II promoter	110	9.99E-36
GO:0006793~phosphorus metabolic process	109	5.16E-24
GO:0006796~phosphate metabolic process	109	5.16E-24
GO:0031328~positive regulation of cellular biosynthetic process	107	5.85E-36
GO:0009891~positive regulation of biosynthetic process	107	2.21E-35
GO:0010033~response to organic substance	103	3.59E-31
GO:0051173~positive regulation of nitrogen compound metabolic process	102	1.03E-34
GO:0010557~positive regulation of macromolecule biosynthetic process	102	3.96E-34
GO:0010628~positive regulation of gene expression	97	7.4E-35
GO:0045935~positive regulation of nucleobase, nucleoside, nucleotide and nucleic acid metabolic process	96	1.53E-31
GO:0010605~negative regulation of macromolecule metabolic process	96	5.61E-26
GO:0016310~phosphorylation	94	4.93E-22
GO:0045941~positive regulation of transcription	92	3.55E-32
GO:0006468~protein amino acid phosphorylation	89	1.24E-24
GO:0008284~positive regulation of cell proliferation	85	2.83E-37
GO:0007049~cell cycle	85	5.6E-18
GO:0051174~regulation of phosphorus metabolic process	84	3.44E-31
GO:0019220~regulation of phosphate metabolic process	84	3.44E-31
GO:0042325~regulation of phosphorylation	83	1.06E-31
GO:0045893~positive regulation of transcription, DNA-dependent	82	3.42E-30
GO:0051254~positive regulation of RNA metabolic process	82	6.23E-30
GO:0008219~cell death	82	2.2E-18
GO:0016265~death	82	3.34E-18
GO:0009890~negative regulation of biosynthetic process	79	1.05E-22
GO:0043069~negative regulation of programmed cell death	77	4.94E-35
GO:0060548~negative regulation of cell death	77	6.04E-35
GO:0045934~negative regulation of nucleobase, nucleoside, nucleotide and nucleic acid metabolic process	77	1.71E-24

GO:0051172~negative regulation of nitrogen compound metabolic process	77	4.07E-24
GO:0010558~negative regulation of macromolecule biosynthetic process	77	1.11E-22
GO:0031327~negative regulation of cellular biosynthetic process	77	5.3E-22
GO:0012501~programmed cell death	77	9.19E-20
GO:0043066~negative regulation of apoptosis	76	1.35E-34
GO:0006928~cell motion	76	6.89E-26
GO:0010629~negative regulation of gene expression	76	3.04E-24
GO:0044093~positive regulation of molecular function	76	3.08E-20
GO:0009719~response to endogenous stimulus	75	1.06E-29
GO:0006915~apoptosis	75	5.82E-19
GO:0009725~response to hormone stimulus	72	4.03E-30
GO:0016481~negative regulation of transcription	71	2.77E-23
GO:0051338~regulation of transferase activity	70	4.03E-28
GO:0045944~positive regulation of transcription from RNA polymerase II promoter	69	2.12E-27
GO:0043085~positive regulation of catalytic activity	69	6.61E-19
GO:0007167~enzyme linked receptor protein signaling pathway	68	8.82E-29
GO:0043549~regulation of kinase activity	68	1.25E-27
GO:0009611~response to wounding	68	7.3E-18
GO:0043065~positive regulation of apoptosis	67	3.85E-22
GO:0043068~positive regulation of programmed cell death	67	5.68E-22
GO:0010942~positive regulation of cell death	67	7.34E-22
GO:0051726~regulation of cell cycle	64	2.31E-26
GO:0045859~regulation of protein kinase activity	64	2.49E-25
GO:0022402~cell cycle process	64	4.15E-14
GO:0042592~homeostatic process	64	7.72E-09
GO:0030182~neuron differentiation	63	5.03E-19
GO:0008285~negative regulation of cell proliferation	61	5.62E-22
GO:0008283~cell proliferation	61	7.7E-18
GO:0045892~negative regulation of transcription, DNA-dependent	59	7.81E-21
GO:0051253~negative regulation of RNA metabolic process	59	1.8E-20
GO:0051094~positive regulation of developmental process	58	1.26E-25
GO:0007243~protein kinase cascade	58	2.65E-19
GO:0032268~regulation of cellular protein metabolic process	58	3.03E-14
GO:0001944~vasculature development	57	3.81E-27
GO:0007155~cell adhesion	57	2.82E-07
GO:0022610~biological adhesion	57	2.89E-07
GO:0001568~blood vessel development	56	8.13E-27
GO:0040008~regulation of growth	55	6.92E-19
GO:0032989~cellular component morphogenesis	54	3.36E-15
GO:0051270~regulation of cell motion	53	1.68E-29
GO:0051674~localization of cell	53	1.55E-19
GO:0048870~cell motility	53	1.55E-19
GO:0043009~chordate embryonic development	53	4.54E-18

GO:0009792~embryonic development ending in birth or egg hatching	53	6.76E-18
GO:0016477~cell migration	52	6.77E-21
GO:0010647~positive regulation of cell communication	52	1.72E-17
GO:0040012~regulation of locomotion	51	1.21E-27
GO:0000902~cell morphogenesis	51	2.82E-15
GO:0009628~response to abiotic stimulus	51	1.07E-14
GO:0035295~tube development	50	8.01E-24
GO:0045597~positive regulation of cell differentiation	50	5.24E-23
GO:0001501~skeletal system development	50	1.1E-16
GO:0030334~regulation of cell migration	49	1.96E-28
GO:0007169~transmembrane receptor protein tyrosine kinase signaling pathway	49	1.38E-22
GO:0030030~cell projection organization	49	1.8E-13
GO:0022403~cell cycle phase	49	1.4E-11
GO:0009967~positive regulation of signal transduction	48	1.11E-16
GO:0048514~blood vessel morphogenesis	47	5.14E-22
GO:0045596~negative regulation of cell differentiation	47	1.43E-21
GO:0006917~induction of apoptosis	47	1.6E-14
GO:0012502~induction of programmed cell death	47	1.79E-14
GO:0000122~negative regulation of transcription from RNA polymerase II promoter	46	4.97E-17
GO:0048666~neuron development	46	5.71E-13
GO:0048878~chemical homeostasis	46	3.39E-07
GO:0032535~regulation of cellular component size	45	6.46E-16
GO:0031399~regulation of protein modification process	45	1.58E-14
GO:0048598~embryonic morphogenesis	45	6.82E-14
GO:0048545~response to steroid hormone stimulus	44	3.94E-21
GO:0006916~anti-apoptosis	44	7.01E-20
GO:0000278~mitotic cell cycle	44	1.58E-10
GO:0033674~positive regulation of kinase activity	43	3.91E-17
GO:0051347~positive regulation of transferase activity	43	1.63E-16
GO:0007389~pattern specification process	43	9.82E-15
GO:0048609~reproductive process in a multicellular organism	43	1.4E-06
GO:0032504~multicellular organism reproduction	43	1.4E-06
GO:0006461~protein complex assembly	43	3.51E-06
GO:0070271~protein complex biogenesis	43	3.51E-06
GO:0002520~immune system development	42	1.49E-13
GO:0042493~response to drug	41	1.16E-16
GO:0007610~behavior	41	3.29E-06
GO:0060284~regulation of cell development	40	1.09E-16
GO:0008361~regulation of cell size	40	1.3E-16
GO:0045860~positive regulation of protein kinase activity	40	2.5E-15
GO:0048534~hemopoietic or lymphoid organ development	40	4.36E-13
GO:0044092~negative regulation of molecular function	40	1.02E-09

GO:0051272~positive regulation of cell motion	39	2.31E-28
GO:0040017~positive regulation of locomotion	39	2.31E-28
GO:0051130~positive regulation of cellular component organization	39	8.55E-18
GO:0000904~cell morphogenesis involved in differentiation	39	2.65E-13
GO:0001775~cell activation	39	4.23E-11
GO:0001525~angiogenesis	38	4.5E-20
GO:0051247~positive regulation of protein metabolic process	38	1.12E-12
GO:0010627~regulation of protein kinase cascade	38	2.38E-12
GO:0031175~neuron projection development	38	5.58E-12
GO:0030335~positive regulation of cell migration	37	1.06E-27
GO:0009991~response to extracellular stimulus	37	2.49E-13
GO:0007423~sensory organ development	37	8.6E-13
GO:0030097~hemopoiesis	37	2.16E-12
GO:0006351~transcription, DNA-dependent	37	1.04E-09
GO:0032774~RNA biosynthetic process	37	1.51E-09
GO:0007010~cytoskeleton organization	37	2.09E-05
GO:0001932~regulation of protein amino acid phosphorylation	36	6.91E-16
GO:0042060~wound healing	36	1.63E-14
GO:0032270~positive regulation of cellular protein metabolic process	36	6.93E-12
GO:0045321~leukocyte activation	36	2.08E-11
GO:0010648~negative regulation of cell communication	36	4.21E-11
GO:0030155~regulation of cell adhesion	35	1.95E-18
GO:0043434~response to peptide hormone stimulus	35	9.34E-17
GO:0001558~regulation of cell growth	35	1.52E-13
GO:0003006~reproductive developmental process	35	7.91E-10
GO:0051301~cell division	35	1.73E-08
GO:0035239~tube morphogenesis	34	1.37E-18
GO:0051960~regulation of nervous system development	34	6.16E-13
GO:0032990~cell part morphogenesis	34	1.68E-09
GO:0007264~small GTPase mediated signal transduction	34	1.29E-07
GO:0051329~interphase of mitotic cell cycle	33	1.22E-20
GO:0051325~interphase	33	3.24E-20
GO:0048729~tissue morphogenesis	33	5.35E-13
GO:0048812~neuron projection morphogenesis	33	5.62E-11
GO:0009968~negative regulation of signal transduction	33	1.5E-10
GO:0048858~cell projection morphogenesis	33	2.14E-09
GO:0048732~gland development	32	7.83E-16
GO:0031667~response to nutrient levels	32	3.36E-11
GO:0003002~regionalization	32	3.36E-11
GO:0050767~regulation of neurogenesis	31	1.86E-12
GO:0001701~in utero embryonic development	31	8.83E-12
GO:0014070~response to organic cyclic substance	30	1.98E-15
GO:0032583~regulation of gene-specific transcription	30	3.5E-14
GO:0045165~cell fate commitment	30	9.57E-14

GO:0070482~response to oxygen levels	30	1.41E-13
GO:0000165~MAPKKK cascade	30	1.41E-10
GO:0007507~heart development	30	5.87E-09
GO:0033043~regulation of organelle organization	30	7.28E-09
GO:0044057~regulation of system process	30	1.26E-05
GO:0046649~lymphocyte activation	29	4.15E-09
GO:0048667~cell morphogenesis involved in neuron differentiation	29	1.25E-08
GO:0060429~epithelium development	29	7.63E-08
GO:0006366~transcription from RNA polymerase II promoter	29	1.46E-07
GO:0051240~positive regulation of multicellular organismal process	29	3.52E-07
GO:0043086~negative regulation of catalytic activity	29	4.45E-06
GO:0051098~regulation of binding	28	4.32E-11
GO:0007409~axonogenesis	28	8.94E-09
GO:0010035~response to inorganic substance	28	3.3E-08
GO:0060341~regulation of cellular localization	28	1.6E-06
GO:0043627~response to estrogen stimulus	27	2.51E-14
GO:0001666~response to hypoxia	27	1.07E-11
GO:0043405~regulation of MAP kinase activity	27	3.58E-11
GO:0044087~regulation of cellular component biogenesis	27	4.22E-11
GO:0032101~regulation of response to external stimulus	27	5.64E-10
GO:0006979~response to oxidative stress	27	1.13E-09
GO:0031401~positive regulation of protein modification process	27	1.92E-08
GO:0009314~response to radiation	27	7.75E-08
GO:0030029~actin filament-based process	27	2.97E-06
GO:0060541~respiratory system development	26	4.16E-13
GO:0007568~aging	26	6.49E-13
GO:0022604~regulation of cell morphogenesis	26	3.93E-11
GO:0007398~ectoderm development	26	2.69E-07
GO:0051050~positive regulation of transport	26	2.29E-06
GO:0030036~actin cytoskeleton organization	26	2.93E-06
GO:0030324~lung development	25	4.16E-13
GO:0030323~respiratory tube development	25	8.41E-13
GO:0045926~negative regulation of growth	25	4.84E-12
GO:0051129~negative regulation of cellular component organization	25	1.31E-09
GO:0007346~regulation of mitotic cell cycle	25	5.37E-09
GO:0040007~growth	25	2.08E-07
GO:0010608~posttranscriptional regulation of gene expression	25	2.83E-06
GO:0048584~positive regulation of response to stimulus	25	1.94E-05
GO:0046777~protein amino acid autophosphorylation	24	1.02E-13
GO:0032868~response to insulin stimulus	24	4.22E-12
GO:0002521~leukocyte differentiation	24	1.34E-09
GO:0045664~regulation of neuron differentiation	24	1.83E-09
GO:0032870~cellular response to hormone stimulus	24	1.83E-09
GO:0032880~regulation of protein localization	24	3.85E-09

GO:0030900~forebrain development	24	2.57E-08
GO:0048568~embryonic organ development	24	2.62E-07
GO:0008544~epidermis development	24	8.84E-07
GO:0007517~muscle organ development	24	9.28E-06
GO:0001763~morphogenesis of a branching structure	23	4.11E-14
GO:0034097~response to cytokine stimulus	23	1.84E-13
GO:0051101~regulation of DNA binding	23	1.55E-09
GO:0051051~negative regulation of transport	23	1.29E-08
GO:0007584~response to nutrient	23	2.56E-08
GO:0009952~anterior/posterior pattern formation	23	2.56E-08
GO:0043062~extracellular structure organization	23	4.05E-07
GO:0000082~G1/S transition of mitotic cell cycle	22	7.04E-16
GO:0048754~branching morphogenesis of a tube	22	2.43E-14
GO:0045786~negative regulation of cell cycle	22	3.04E-12
GO:0010551~regulation of specific transcription from RNA polymerase II promoter	22	6.58E-11
GO:0045792~negative regulation of cell size	22	1.86E-10
GO:0043408~regulation of MAPKKK cascade	22	1.22E-09
GO:0060348~bone development	22	1.18E-08
GO:0031099~regeneration	21	1.02E-12
GO:0050678~regulation of epithelial cell proliferation	21	1.85E-12
GO:0031344~regulation of cell projection organization	21	1.68E-10
GO:0001503~ossification	21	1.9E-08
GO:0051241~negative regulation of multicellular organismal process	21	6.65E-06
GO:0051345~positive regulation of hydrolase activity	21	2.46E-05
GO:0000079~regulation of cyclin-dependent protein kinase activity	20	7.15E-14
GO:0043193~positive regulation of gene-specific transcription	20	8.12E-10
GO:0043523~regulation of neuron apoptosis	20	1.5E-09
GO:0030308~negative regulation of cell growth	20	2.22E-09
GO:0051348~negative regulation of transferase activity	20	4.7E-09
GO:0042327~positive regulation of phosphorylation	20	5.63E-09
GO:0010562~positive regulation of phosphorus metabolic process	20	9.57E-09
GO:0045937~positive regulation of phosphate metabolic process	20	9.57E-09
GO:0007050~cell cycle arrest	20	1.59E-08
GO:0019216~regulation of lipid metabolic process	20	6.54E-08
GO:0045137~development of primary sexual characteristics	20	5.04E-07
GO:0051493~regulation of cytoskeleton organization	20	1.47E-06
GO:0007548~sex differentiation	20	7.13E-06
GO:0010720~positive regulation of cell development	19	9.85E-11
GO:0010769~regulation of cell morphogenesis involved in differentiation	19	6.98E-10
GO:0009894~regulation of catabolic process	19	2.92E-08
GO:0048585~negative regulation of response to stimulus	19	5.65E-08
GO:0021700~developmental maturation	19	6.63E-08
GO:0002009~morphogenesis of an epithelium	19	6.63E-08

GO:0043406~positive regulation of MAP kinase activity	19	7.76E-08
GO:0007178~transmembrane receptor protein serine/threonine kinase signaling pathway	19	9.07E-08
GO:0060173~limb development	19	9.07E-08
GO:0048736~appendage development	19	9.07E-08
GO:0007265~Ras protein signal transduction	19	1.23E-07
GO:0048705~skeletal system morphogenesis	19	3.38E-07
GO:0070201~regulation of establishment of protein localization	19	1.09E-06
GO:0048608~reproductive structure development	19	2E-06
GO:0030879~mammary gland development	18	1.83E-10
GO:0010638~positive regulation of organelle organization	18	1.78E-08
GO:0001934~positive regulation of protein amino acid phosphorylation	18	5.31E-08
GO:0033673~negative regulation of kinase activity	18	6.32E-08
GO:0043583~ear development	18	1.45E-07
GO:0035107~appendage morphogenesis	18	2.69E-07
GO:0035108~limb morphogenesis	18	2.69E-07
GO:0051090~regulation of transcription factor activity	18	4.84E-07
GO:0032844~regulation of homeostatic process	18	2.1E-06
GO:0032147~activation of protein kinase activity	18	2.1E-06
GO:0048511~rhythmic process	18	1.04E-05
GO:0016055~Wnt receptor signaling pathway	18	1.75E-05
GO:0048562~embryonic organ morphogenesis	18	1.75E-05
GO:0009612~response to mechanical stimulus	17	2.47E-10
GO:0032869~cellular response to insulin stimulus	17	5.63E-09
GO:0045637~regulation of myeloid cell differentiation	17	8.83E-09
GO:0000302~response to reactive oxygen species	17	2.54E-08
GO:0031960~response to corticosteroid stimulus	17	1.62E-07
GO:0006469~negative regulation of protein kinase activity	17	2.27E-07
GO:0030098~lymphocyte differentiation	17	2.41E-06
GO:0030198~extracellular matrix organization	17	2.75E-06
GO:0007411~axon guidance	17	4.03E-06
GO:0007611~learning or memory	17	6.55E-06
GO:0008406~gonad development	17	7.37E-06
GO:0051223~regulation of protein transport	17	9.29E-06
GO:0045765~regulation of angiogenesis	16	1.42E-08
GO:0051216~cartilage development	16	1.43E-07
GO:0030278~regulation of ossification	16	2.95E-07
GO:0042770~DNA damage response, signal transduction	16	4.17E-07
GO:0035113~embryonic appendage morphogenesis	16	1.28E-06
GO:0030326~embryonic limb morphogenesis	16	1.28E-06
GO:0080135~regulation of cellular response to stress	16	1.12E-05
GO:0007565~female pregnancy	16	2.5E-05
GO:0048660~regulation of smooth muscle cell proliferation	15	1.31E-09
GO:0010810~regulation of cell-substrate adhesion	15	1.31E-09

GO:0008629~induction of apoptosis by intracellular signals	15	1.33E-08
GO:0045787~positive regulation of cell cycle	15	2.82E-08
GO:0010552~positive regulation of specific transcription from RNA polymerase II promoter	15	2.82E-08
GO:0045785~positive regulation of cell adhesion	15	5.71E-08
GO:0031329~regulation of cellular catabolic process	15	7.15E-08
GO:0033273~response to vitamin	15	2.05E-07
GO:0010975~regulation of neuron projection development	15	4.43E-07
GO:0032386~regulation of intracellular transport	15	5.32E-07
GO:0040029~regulation of gene expression, epigenetic	15	1.5E-06
GO:0043281~regulation of caspase activity	15	2.06E-06
GO:0045927~positive regulation of growth	15	2.41E-06
GO:0052548~regulation of endopeptidase activity	15	3.27E-06
GO:0052547~regulation of peptidase activity	15	5.84E-06
GO:0048589~developmental growth	15	6.72E-06
GO:0000075~cell cycle checkpoint	15	1.15E-05
GO:0030099~myeloid cell differentiation	15	1.48E-05
GO:0008637~apoptotic mitochondrial changes	14	4.9E-11
GO:0007162~negative regulation of cell adhesion	14	5.63E-09
GO:0031346~positive regulation of cell projection organization	14	1.87E-08
GO:0043392~negative regulation of DNA binding	14	5.49E-08
GO:0046822~regulation of nucleocytoplasmic transport	14	2.83E-07
GO:0050769~positive regulation of neurogenesis	14	3.5E-07
GO:0051100~negative regulation of binding	14	3.5E-07
GO:0007179~transforming growth factor beta receptor signaling pathway	14	4.31E-07
GO:0042063~gliogenesis	14	1.14E-06
GO:0060562~epithelial tube morphogenesis	14	1.64E-06
GO:0030522~intracellular receptor-mediated signaling pathway	14	6.16E-06
GO:0042113~B cell activation	14	7.16E-06
GO:0032496~response to lipopolysaccharide	14	8.32E-06
GO:0051384~response to glucocorticoid stimulus	14	9.63E-06
GO:0001952~regulation of cell-matrix adhesion	13	1.2E-10
GO:0048661~positive regulation of smooth muscle cell proliferation	13	8.44E-10
GO:0008286~insulin receptor signaling pathway	13	8.93E-09
GO:0042306~regulation of protein import into nucleus	13	7.91E-08
GO:0042326~negative regulation of phosphorylation	13	1.04E-07
GO:0045834~positive regulation of lipid metabolic process	13	1.36E-07
GO:0010563~negative regulation of phosphorus metabolic process	13	2.26E-07
GO:0032582~negative regulation of gene-specific transcription	13	2.26E-07
GO:0045936~negative regulation of phosphate metabolic process	13	2.26E-07
GO:0009896~positive regulation of catabolic process	13	2.89E-07
GO:0045444~fat cell differentiation	13	7.24E-07
GO:0033157~regulation of intracellular protein transport	13	7.24E-07
GO:0032355~response to estradiol stimulus	13	8.98E-07

GO:0042476~odontogenesis	13	8.98E-07
GO:0050770~regulation of axonogenesis	13	1.66E-06
GO:0001890~placenta development	13	2.96E-06
GO:0009636~response to toxin	13	3.55E-06
GO:0031668~cellular response to extracellular stimulus	13	6.01E-06
GO:0032103~positive regulation of response to external stimulus	13	6.01E-06
GO:0045471~response to ethanol	13	6.01E-06
GO:0035270~endocrine system development	13	1.35E-05
GO:0007229~integrin-mediated signaling pathway	13	1.57E-05
GO:0010906~regulation of glucose metabolic process	12	5.44E-08
GO:0010675~regulation of cellular carbohydrate metabolic process	12	1.41E-07
GO:0006109~regulation of carbohydrate metabolic process	12	1.9E-07
GO:0050679~positive regulation of epithelial cell proliferation	12	3.33E-07
GO:0050795~regulation of behavior	12	1.18E-06
GO:0030183~B cell differentiation	12	1.85E-06
GO:0019217~regulation of fatty acid metabolic process	12	2.31E-06
GO:0016202~regulation of striated muscle tissue development	12	2.86E-06
GO:0048634~regulation of muscle development	12	3.51E-06
GO:0010565~regulation of cellular ketone metabolic process	12	1.1E-05
GO:0034330~cell junction organization	12	1.1E-05
GO:0001836~release of cytochrome c from mitochondria	11	1.91E-09
GO:0031100~organ regeneration	11	2.4E-08
GO:0050921~positive regulation of chemotaxis	11	8.15E-08
GO:0050920~regulation of chemotaxis	11	1.68E-07
GO:0001936~regulation of endothelial cell proliferation	11	2.36E-07
GO:0007569~cell aging	11	3.27E-07
GO:0048520~positive regulation of behavior	11	4.47E-07
GO:0031331~positive regulation of cellular catabolic process	11	8.07E-07
GO:0010553~negative regulation of specific transcription from RNA polymerase II promoter	11	2.98E-06
GO:0022405~hair cycle process	11	3.78E-06
GO:0022404~molting cycle process	11	3.78E-06
GO:0001942~hair follicle development	11	3.78E-06
GO:0042303~molting cycle	11	4.75E-06
GO:0042633~hair cycle	11	4.75E-06
GO:0051495~positive regulation of cytoskeleton organization	11	7.36E-06
GO:0043433~negative regulation of transcription factor activity	11	7.36E-06
GO:0030111~regulation of Wnt receptor signaling pathway	11	9.08E-06
GO:0018108~peptidyl-tyrosine phosphorylation	11	9.08E-06
GO:0010721~negative regulation of cell development	11	1.36E-05
GO:0045793~positive regulation of cell size	11	1.36E-05
GO:0018212~peptidyl-tyrosine modification	11	1.36E-05
GO:0051385~response to mineralocorticoid stimulus	10	2.36E-08
GO:0001569~patterning of blood vessels	10	3.99E-08

GO:0045639~positive regulation of myeloid cell differentiation	10	3.43E-06
GO:0046324~regulation of glucose import	10	3.43E-06
GO:0010827~regulation of glucose transport	10	4.5E-06
GO:0051781~positive regulation of cell division	10	1.53E-05
GO:0051147~regulation of muscle cell differentiation	10	1.53E-05
GO:0051341~regulation of oxidoreductase activity	10	1.9E-05
GO:0046626~regulation of insulin receptor signaling pathway	9	2.76E-07
GO:0010907~positive regulation of glucose metabolic process	9	2.76E-07
GO:0032570~response to progesterone stimulus	9	4.44E-07
GO:0010676~positive regulation of cellular carbohydrate metabolic process	9	4.44E-07
GO:0045913~positive regulation of carbohydrate metabolic process	9	4.44E-07
GO:0010594~regulation of endothelial cell migration	9	3.2E-06
GO:0048730~epidermis morphogenesis	9	3.2E-06
GO:0000186~activation of MAPKK activity	9	3.2E-06
GO:0019827~stem cell maintenance	9	3.2E-06
GO:0048864~stem cell development	9	4.47E-06
GO:0046320~regulation of fatty acid oxidation	9	8.29E-06
GO:0030856~regulation of epithelial cell differentiation	9	1.11E-05
GO:0043467~regulation of generation of precursor metabolites and energy	9	1.46E-05
GO:0048863~stem cell differentiation	9	2.44E-05
GO:0001933~negative regulation of protein amino acid phosphorylation	9	2.44E-05
GO:0051412~response to corticosterone stimulus	8	1.16E-06
GO:0051402~neuron apoptosis	8	4.61E-06
GO:0043255~regulation of carbohydrate biosynthetic process	8	9.93E-06
GO:0043542~endothelial cell migration	8	1.41E-05
GO:0014065~phosphoinositide 3-kinase cascade	7	2.41E-08
GO:0033627~cell adhesion mediated by integrin	7	2.41E-08
GO:0045737~positive regulation of cyclin-dependent protein kinase activity	7	9.31E-08
GO:0010812~negative regulation of cell-substrate adhesion	7	1.39E-06
GO:0002687~positive regulation of leukocyte migration	7	1.31E-05
GO:0046627~negative regulation of insulin receptor signaling pathway	7	1.31E-05
GO:0043535~regulation of blood vessel endothelial cell migration	7	2.03E-05
GO:0046321~positive regulation of fatty acid oxidation	6	4.64E-06
GO:0031998~regulation of fatty acid beta-oxidation	6	4.64E-06
GO:0033158~regulation of protein import into nucleus, translocation	6	1.01E-05
GO:0043550~regulation of lipid kinase activity	6	1.96E-05
GO:0001953~negative regulation of cell-matrix adhesion	6	1.96E-05
**Molecular function**		
GO:0003677~DNA binding	149	6.86E-11
GO:0030528~transcription regulator activity	145	1.57E-26
GO:0000166~nucleotide binding	130	7.88E-07
GO:0017076~purine nucleotide binding	119	7.57E-08
GO:0032555~purine ribonucleotide binding	116	4.45E-08
GO:0032553~ribonucleotide binding	116	4.45E-08

GO:0003700~transcription factor activity	105	1.86E-22
GO:0001882~nucleoside binding	105	5.43E-08
GO:0001883~purine nucleoside binding	104	7.36E-08
GO:0030554~adenyl nucleotide binding	101	2.42E-07
GO:0005524~ATP binding	98	7.36E-08
GO:0032559~adenyl ribonucleotide binding	98	1.4E-07
GO:0043565~sequence-specific DNA binding	80	2.74E-22
GO:0004672~protein kinase activity	78	4.34E-21
GO:0042802~identical protein binding	70	4.64E-15
GO:0019899~enzyme binding	68	1.33E-18
GO:0008134~transcription factor binding	67	1.98E-18
GO:0046983~protein dimerization activity	66	1.29E-16
GO:0016563~transcription activator activity	62	3.09E-20
GO:0004674~protein serine/threonine kinase activity	47	3.13E-10
GO:0016564~transcription repressor activity	42	7.56E-12
GO:0046982~protein heterodimerization activity	36	1.36E-13
GO:0003712~transcription cofactor activity	36	5.73E-07
GO:0042803~protein homodimerization activity	35	2.35E-07
GO:0003702~RNA polymerase II transcription factor activity	34	3.01E-10
GO:0019900~kinase binding	32	1.6E-12
GO:0032403~protein complex binding	31	9.17E-11
GO:0004713~protein tyrosine kinase activity	29	3.76E-11
GO:0019901~protein kinase binding	26	3.57E-10
GO:0043566~structure-specific DNA binding	24	7.35E-09
GO:0003690~double-stranded DNA binding	23	1.18E-11
GO:0003682~chromatin binding	22	2.99E-07
GO:0019838~growth factor binding	20	1.67E-08
GO:0004714~transmembrane receptor protein tyrosine kinase activity	19	4.31E-11
GO:0010843~promoter binding	17	2.56E-10
GO:0019207~kinase regulator activity	16	2.48E-06
GO:0046332~SMAD binding	13	1.12E-07
GO:0005178~integrin binding	12	1.32E-05
GO:0005518~collagen binding	10	6.55E-06
GO:0031625~ubiquitin protein ligase binding	10	6.55E-06
GO:0003705~RNA polymerase II transcription factor activity, enhancer binding	10	1.33E-05
GO:0003704~specific RNA polymerase II transcription factor activity	10	3.08E-05
GO:0016538~cyclin-dependent protein kinase regulator activity	7	2.76E-05
GO:0016303~1-phosphatidylinositol-3-kinase activity	6	3.19E-05
**KEGG pathway**		
hsa05200:Pathways in cancer	110	7.49E-56
hsa04510:Focal adhesion	59	4.54E-25
hsa04010:MAPK signaling pathway	49	6.04E-12
hsa05215:Prostate cancer	44	2.96E-29

hsa05212:Pancreatic cancer	43	4.4E-33
hsa04810:Regulation of actin cytoskeleton	43	9E-12
hsa05220:Chronic myeloid leukemia	42	9.78E-31
hsa05210:Colorectal cancer	42	3.84E-28
hsa04062:Chemokine signaling pathway	41	1.41E-12
hsa04722:Neurotrophin signaling pathway	40	1.82E-18
hsa05222:Small cell lung cancer	37	2.38E-22
hsa05218:Melanoma	34	6.21E-22
hsa04520:Adherens junction	34	1.47E-20
hsa04110:Cell cycle	34	2.94E-13
hsa05211:Renal cell carcinoma	33	5.09E-21
hsa04012:ErbB signaling pathway	33	1.37E-17
hsa04310:Wnt signaling pathway	33	3.76E-10
hsa05214:Glioma	32	1.34E-21
hsa04910:Insulin signaling pathway	30	1.89E-09
hsa05219:Bladder cancer	29	1.2E-24
hsa05223:Non-small cell lung cancer	29	2.1E-20
hsa04660:T cell receptor signaling pathway	28	1.88E-10
hsa05213:Endometrial cancer	27	1.68E-18
hsa04666:Fc gamma R-mediated phagocytosis	27	4.62E-11
hsa04670:Leukocyte transendothelial migration	27	7.73E-09
hsa04360:Axon guidance	26	2.23E-07
hsa04370:VEGF signaling pathway	25	6.64E-12
hsa05221:Acute myeloid leukemia	24	9.76E-14
hsa04350:TGF-beta signaling pathway	24	1.35E-09
hsa04914:Progesterone-mediated oocyte maturation	23	6.14E-09
hsa04210:Apoptosis	23	7.76E-09
hsa04115:p53 signaling pathway	22	3.08E-10
hsa04662:B cell receptor signaling pathway	21	1.42E-08
hsa04620:Toll-like receptor signaling pathway	21	2.66E-06
hsa04150:mTOR signaling pathway	20	8.14E-11
hsa04664:Fc epsilon RI signaling pathway	19	8.19E-07
hsa04916:Melanogenesis	19	2.91E-05
hsa05120:Epithelial cell signaling in Helicobacter pylori infection	17	2.58E-06
hsa04930:Type II diabetes mellitus	16	6.99E-08
hsa04920:Adipocytokine signaling pathway	16	1.03E-05
hsa05216:Thyroid cancer	12	5.66E-07
hsa04960:Aldosterone-regulated sodium reabsorption	12	2.63E-05

Supplementary table S3 | Shared annotation terms for target proteins in group1, group2, group3 and their ranks in terms for all targets.

Term	P-value	Rank
**Biological process**		
GO:0042127~regulation of cell proliferation	3.18E-60	1
GO:0043067~regulation of programmed cell death	5.28E-51	2
GO:0010941~regulation of cell death	8.32E-51	3
GO:0042981~regulation of apoptosis	6.17E-50	4
GO:0010604~positive regulation of macromolecule metabolic process	2.80E-39	5
GO:0008284~positive regulation of cell proliferation	2.83E-37	6
GO:0031328~positive regulation of cellular biosynthetic process	5.85E-36	7
GO:0006357~regulation of transcription from RNA polymerase II promoter	9.99E-36	8
GO:0009891~positive regulation of biosynthetic process	2.21E-35	9
GO:0043069~negative regulation of programmed cell death	4.94E-35	10
GO:0060548~negative regulation of cell death	6.04E-35	11
GO:0010628~positive regulation of gene expression	7.40E-35	12
GO:0051173~positive regulation of nitrogen compound metabolic process	1.03E-34	13
GO:0043066~negative regulation of apoptosis	1.35E-34	14
GO:0010557~positive regulation of macromolecule biosynthetic process	3.96E-34	15
GO:0045941~positive regulation of transcription	3.55E-32	16
GO:0042325~regulation of phosphorylation	1.06E-31	17
GO:0045935~positive regulation of nucleobase, nucleoside, nucleotide and nucleic acid metabolic process	1.53E-31	18
GO:0051174~regulation of phosphorus metabolic process	3.44E-31	19
GO:0019220~regulation of phosphate metabolic process	3.44E-31	20
GO:0010033~response to organic substance	3.59E-31	21
GO:0045893~positive regulation of transcription, DNA-dependent	3.42E-30	22
GO:0009725~response to hormone stimulus	4.03E-30	23
GO:0051254~positive regulation of RNA metabolic process	6.23E-30	24
GO:0009719~response to endogenous stimulus	1.06E-29	25
GO:0051270~regulation of cell motion	1.68E-29	26
GO:0030334~regulation of cell migration	1.96E-28	28
GO:0051272~positive regulation of cell motion	2.31E-28	29
GO:0040017~positive regulation of locomotion	2.31E-28	30
GO:0051338~regulation of transferase activity	4.03E-28	31
GO:0030335~positive regulation of cell migration	1.06E-27	32
GO:0040012~regulation of locomotion	1.21E-27	33
GO:0043549~regulation of kinase activity	1.25E-27	34
GO:0045944~positive regulation of transcription from RNA polymerase II promoter	2.12E-27	35
GO:0051726~regulation of cell cycle	2.31E-26	39
GO:0051252~regulation of RNA metabolic process	1.81E-25	43
GO:0045859~regulation of protein kinase activity	2.49E-25	44
GO:0006355~regulation of transcription, DNA-dependent	1.26E-24	46

GO:0010629~negative regulation of gene expression	3.04E-24	48
GO:0045449~regulation of transcription	6.22E-24	52
GO:0035295~tube development	8.01E-24	53
GO:0008285~negative regulation of cell proliferation	5.62E-22	63
GO:0048545~response to steroid hormone stimulus	3.94E-21	67
GO:0030182~neuron differentiation	5.03E-19	80
GO:0043085~positive regulation of catalytic activity	6.61E-19	82
GO:0035239~tube morphogenesis	1.37E-18	84
GO:0007049~cell cycle	5.60E-18	89
GO:0008283~cell proliferation	7.70E-18	92
GO:0009628~response to abiotic stimulus	1.07E-14	113
GO:0031399~regulation of protein modification process	1.58E-14	114
**Molecular function**		
GO:0030528~transcription regulator activity	1.57E-26	1
GO:0003700~transcription factor activity	1.86E-22	2
GO:0016563~transcription activator activity	3.09E-20	5
GO:0019899~enzyme binding	1.33E-18	6
**KEGG pathway**		
hsa05200:Pathways in cancer	7.49E-56	1
hsa05212:Pancreatic cancer	4.40E-33	2
hsa05220:Chronic myeloid leukemia	9.78E-31	3
hsa05215:Prostate cancer	2.96E-29	4
hsa05210:Colorectal cancer	3.84E-28	5
hsa05219:Bladder cancer	1.20E-24	7
hsa05222:Small cell lung cancer	2.38E-22	8
